# Supplementary material for: Heterochromatin-dependent transcription links the PRC2 complex to small RNA-mediated DNA elimination
Source: EMBO Rep. 2024 Nov 29;26(1):273–96. doi: 10.1038/s44319-024-00332-1 (PMC11723920; doi:10.1038/s44319-024-00332-1)
Supplement: Supplementary file 1 — Appendix [file 44319_2024_332_MOESM1_ESM.pdf]

Appendix

Heterochromatin-dependent transcription links the PRC2 complex to small RNA-mediated DNA elimination

Therese Solberg[1,2,3,*], Chundi Wang[1,4,5], Ryuma Matsubara[1,6] , Zhiwei Wen[5] and Mariusz Nowacki[1,*]

[1] Institute of Cell Biology, University of Bern, Baltzerstrasse 4, 3012 Bern, Switzerland

[2] Department of Molecular Biology, Keio University School of Medicine, 160-8582 Tokyo, Japan

[3] Human Biology Microbiome Quantum Research Center (WPI-Bio2Q), Keio University, 108-8345 Tokyo, Japan

[4] Institute of Evolution & Marine Biodiversity, Ocean University of China, Qingdao 266003, China

[5] Laboratory of Marine Protozoan Biodiversity & Evolution, Marine College, Shandong University, Weihai 264209, China

[6] Isotope Science Center, The University of Tokyo, 113-0032 Tokyo, Japan

* Correspondence to: mariusz.nowacki@unibe.ch, therese.solberg@keio.jp

List of Appendix Figures and Tables

Appendix Figures

Figure S1. Phylogenetic relationship of *P. tetraurelia* CDs. Page 3

Figure S2. Localization of Fire1 and Fire2-GFP during new MAC development. Page 4

Figure S3. Localization of May1-GFP during new MAC development. Page 5

Figure S4. Localization of May1-GFP in the absence of PRC2. Page 6

Figure S5. Retained IESs after Fire and May silencing (long IESs). Page 7

Figure S6. Retained IESs after Fire and May silencing (short IESs). Page 8

Figure S7. Shared IES retention between TFIIS4 and Fire1/2 silencing. Page 9

Figure S8. Nucleosome density histograms for various IES subsets. Page 10

Appendix Tables

Table S1. *T. thermophila* CD sequences. Page 11

Table S2. Alignment of *P. tetraurelia* CDs. Page 12

Table S3. Sequence of codon-optimized May1-His for *E. coli* protein expression. Page 13

Table S4. Histone peptides used in this study. Page 14

Appendix Figures

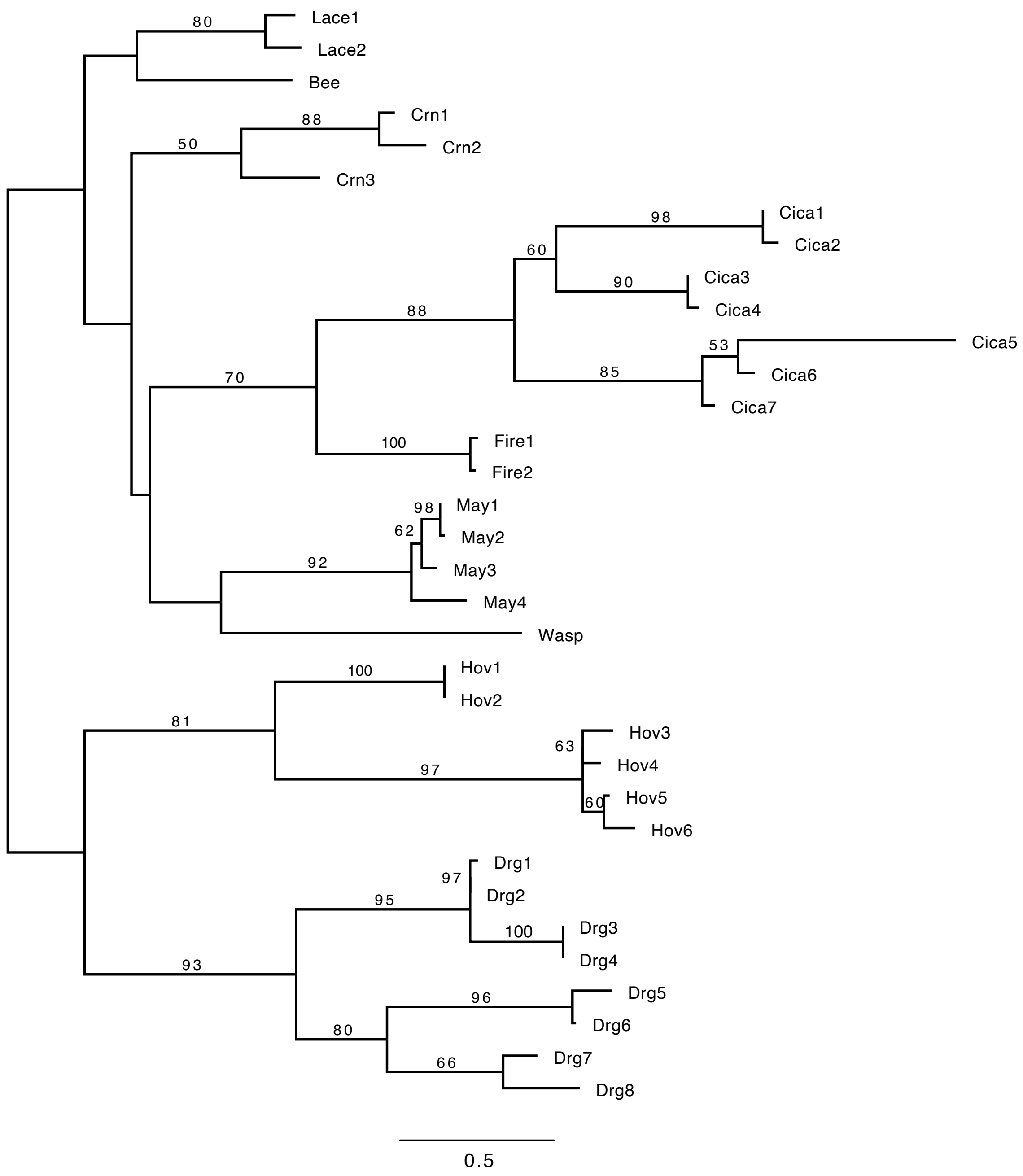

Appendix Figure S1. Phylogenetic relationship of *P. tetraurelia* CDs.

The ML phylogenetic tree was reconstructed with 100 bootstrap replicates using CD sequences identified from InterProScan predictions, a list of which can be found in Dataset EV1. Supports equal or greater than 50 are shown. Related to Figure 1.

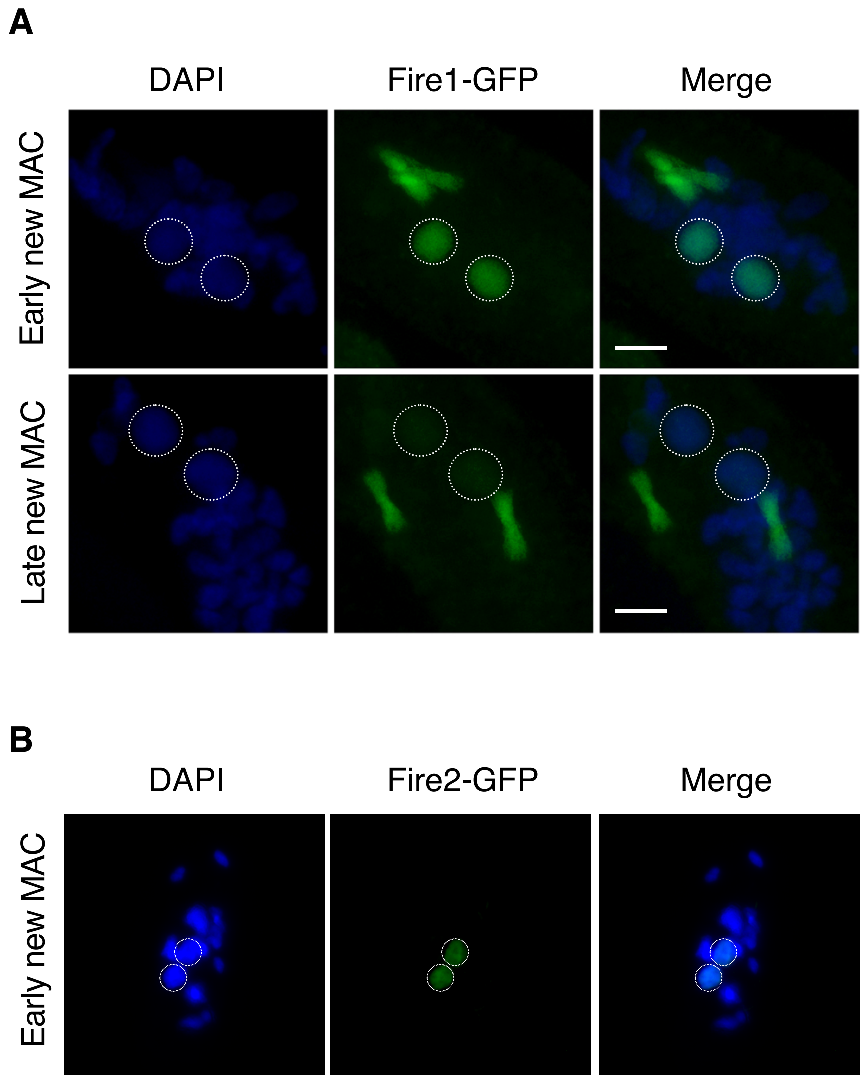

Appendix Figure S2. Localization of Fire1 and Fire2-GFP during new MAC development. **(A)** Localization of Fire1-GFP in early and late new MAC development. Scale bar: 10 μm. **(B)** Localization of Fire2-GFP in early new MAC development. Related to Figure 2.

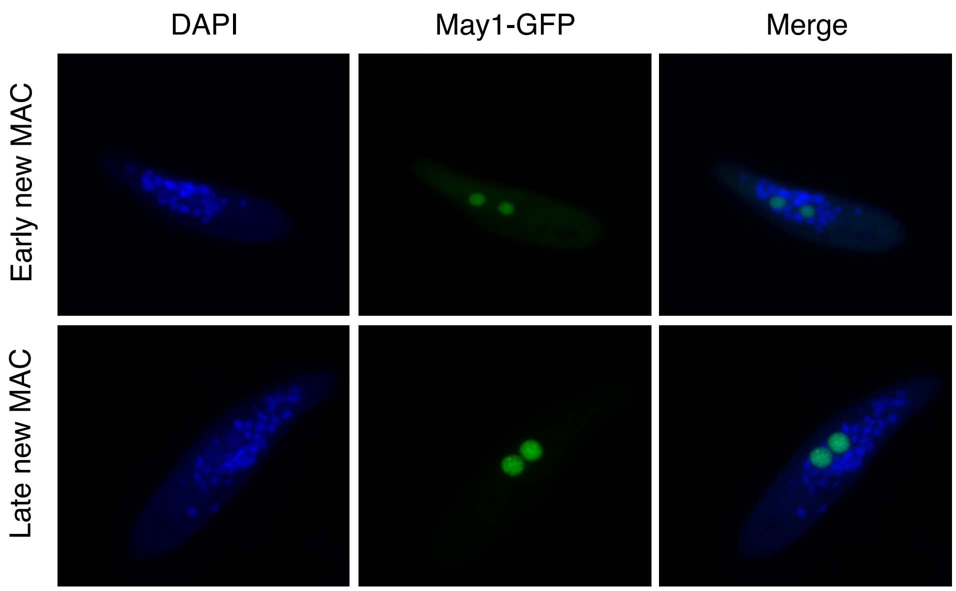

Appendix Figure S3. Localization of May1-GFPduring new MAC development.
Localization of May1-GFP from early new MAC development (top) to post-karyonidal division (bottom). The signal is at first uniform, before forming nuclear condensates. Related to Figure 2.

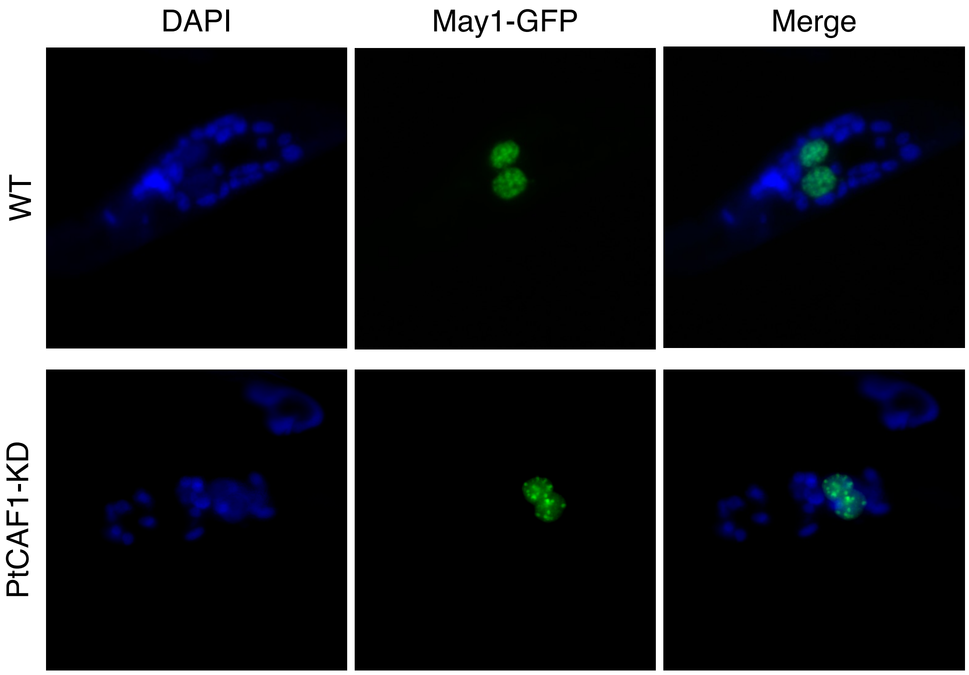

Appendix Figure S4. Localization of May1-GFP in the absence of PRC2.
PRC2 depletion is achieved through silencing of the PtCAF1 subunit, which renders the complex unstable (Wang *et al*, 2022). Note that the top panel is the same as in Figure 2D. Related to Figure 2.

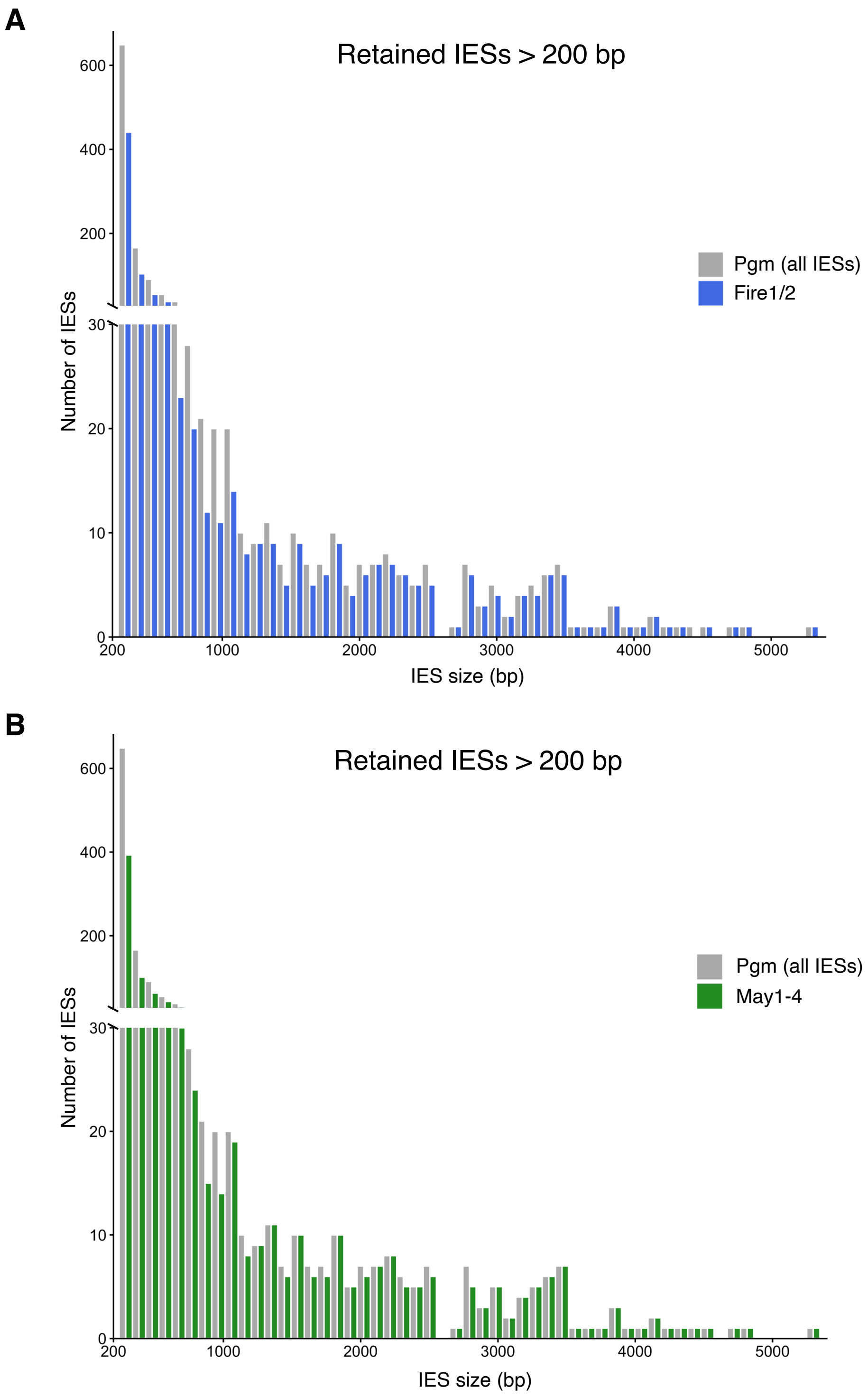

Appendix Figure S5. Retained IESs after Fire and May silencing (long IESs).
Size distribution of retained IESs longer than 200 bp (IRS > 0.1) after Fire1/2 silencing **(A),** or May1-4 silencing **(B)**, in relation to all IESs (Pgm silencing). Note the discontinuous y axis. Related to Figure 3 and Appendix Figure S6.

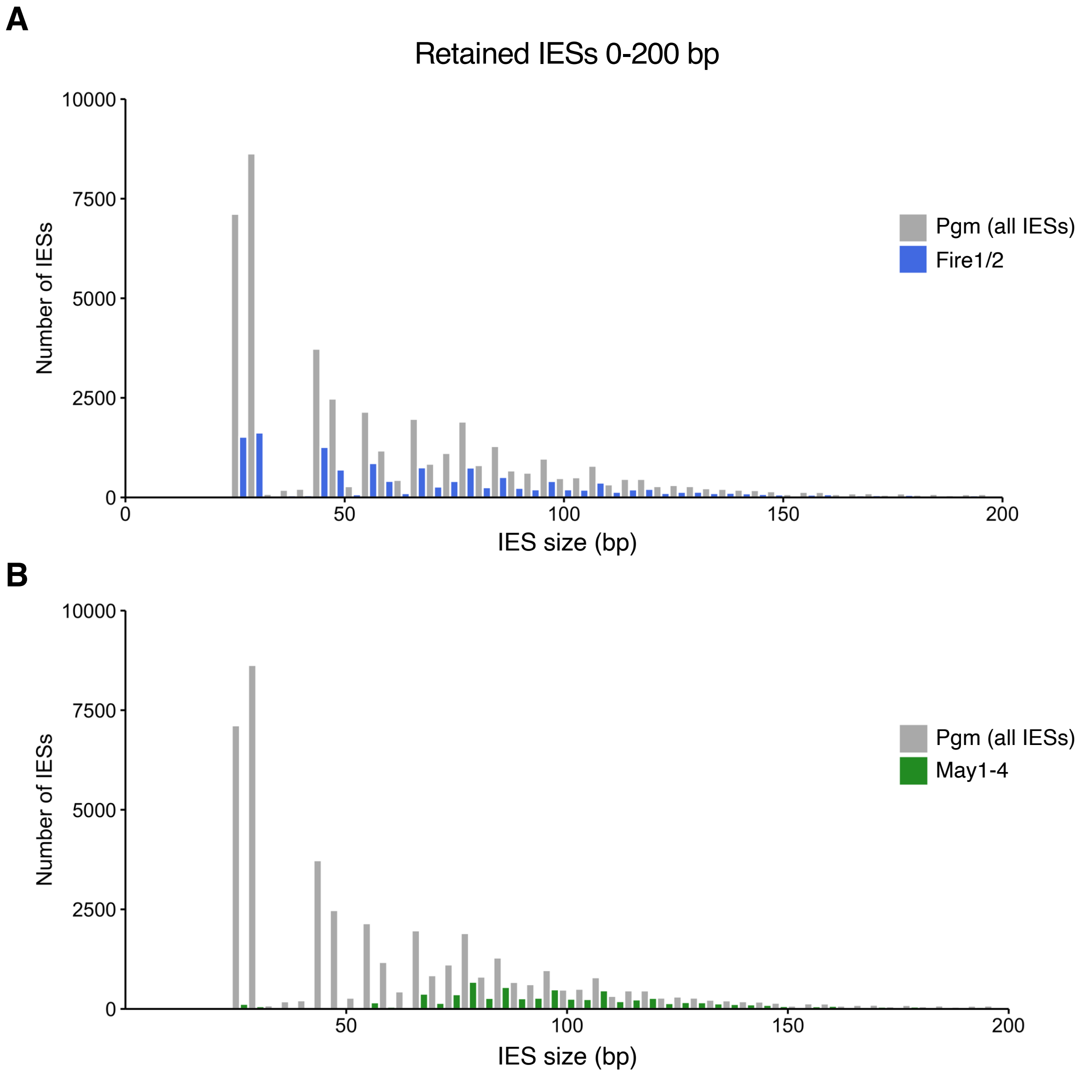

Appendix Figure S6. Retained IESs after Fire and May silencing (short IESs).

Size distribution of retained IESs shorter than 200 bp (IRS > 0.1) after Fire1/2 silencing **(A)**, or May1-4 silencing **(B)**, in relation to all IESs (Pgm silencing). Related to Figure 3 and Appendix Figure S5.

A

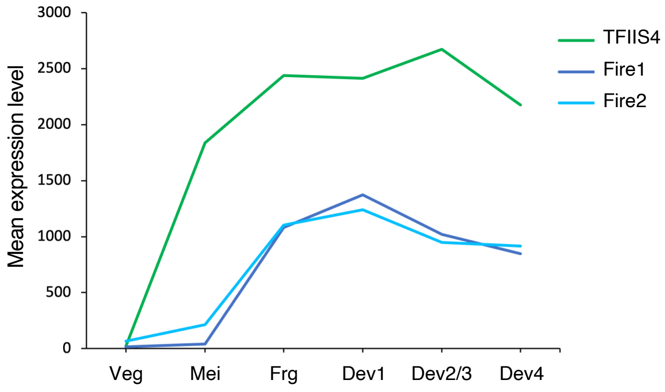

B

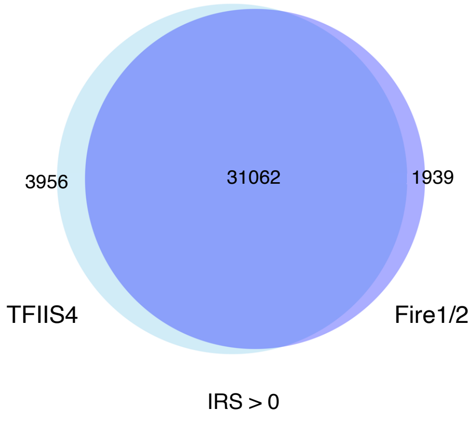

Appendix Figure S7. Shared IES retention between TFIIS4 and Fire1/2 silencing.

(A) Expression profiles of each gene in each subfamily, generated with published DESeq2-normalized RNA-seq counts retrieved from the *Paramecium* Database (Arnaiz *et al*, 2020; Arnaiz *et al*, 2017)**.** The horizontal axis denotes developmental stages from early to late stage of autogamy, as defined in the *Paramecium* Database. Veg: vegetative, Mei: MIC meiosis, Frg: maternal MAC fragmentation, Dev1: early new MAC development, Dev2/3: intermediate new MAC development, Dev4: late new MAC development. **(B)** Venn diagram depicting shared IES retention between TFIIS4 and Fire1/2 silencing, for all retained IESs (IRS > 0). Related to Figure 4.

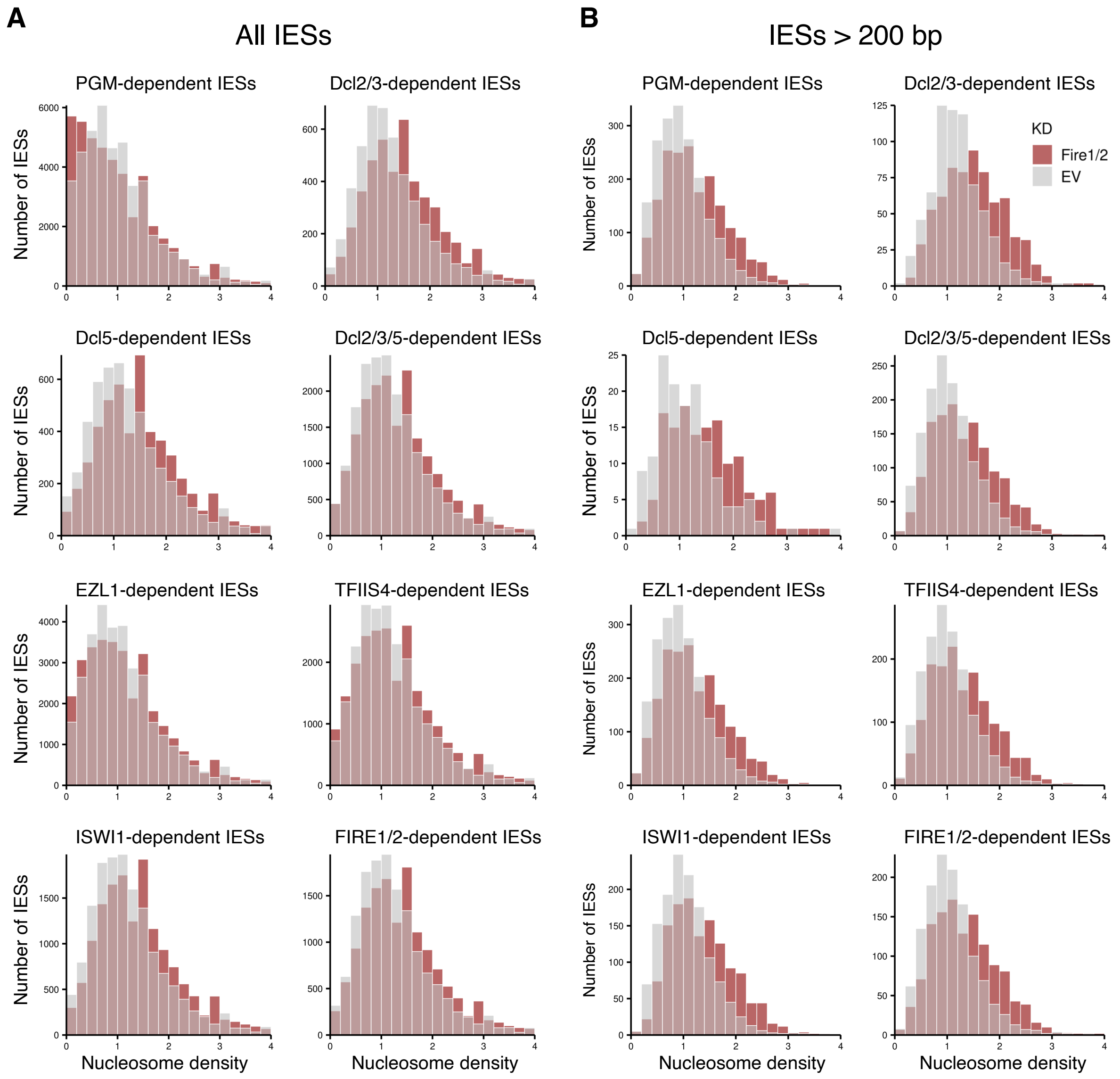

Appendix Figure S8. Nucleosome density histograms for various IES subsets.

(A, B) Normalized nucleosome density histograms for IESs. Nucleosome density is defined as a dimensionless value (Wang *et al.*, 2022). **(A)** Histograms depicting nucleosome density for IES subsets sensitive to the given knockdown (IRS > 0.1), without IES size constraints. **(B)** Histograms with additional cutoff of IESs by size (larger than 200 bp). Related to Figure 5.

Appendix Tables

Appendix Table S1. *Tetrahymena thermophila* CD sequences.

CD sequences were retrieved from (Wiley *et al*, 2018) and used as queries to identify *P. tetraurelia* chromodomain proteins using Blastp. For chromodomain proteins with two CDs, the CDs are numbered 1 and 2. Related to Figure 1.

Name	***Sequence***
Hhp1	VYEVENIIGHRKKGSQIEYHIQWKGYSLKQATYEPAKNILDKNMLKKYQQKHL
Hpl1	EYNVEYLYGKKFENGQIKYCVKWENYTFEESSFEPVENLENVVYNMRGFERKMS
Hpl2_1	EYEVEKIVDKKIENGQIFYKVKWKGWDSTYNTWEPENNLFRVSEMIEEYEASKK
Hpl2_2	EYIFEAILDKRQLEGQEVEYLLKFSNHDKPEWELISNLESIEEEIKEYEEGLK
Hpl3	YYEVESIQKIRSFGKYDLRFYIKWKGWEDTDNTWEPFQNVRNCYFNLKEFYDKYN
Hpl4_1	IFEIESILEKRIHPKTGKTEFLVQWKQWPDDPTWEPAQNIQSFLNNNNQKKK
Hpl4_2	IEPIYKILDKQEVENTIYYLILFYDEKLPQWVKQEKLIGYEDEILEYEKQII
Hpl5	KFEILAILDKRANPKNGKIEYLVQWKQWPLDYTWEPKKNIQNFIDQNELFVDDED
Hpl6	FYIVEKILDYKKIKNRDLFLVKWEGYEELTWEPKSNLSNVKQLVENFLKTLK
Hpl7_1	LYEPEKILDKKVNQSGTSYLVKWKGFRASQATWEFYKNISHCNWLFEDWEEQQN
Hpl7_2	KIKNMDRIIDKKYVNSKVFYLVSLKNGGEDVWIRRKALMLDHADKISEYEKSDQ
Pdd1_1	QYEVEKILDSRFNPKTKQKEYLVKWENWPIEDSTWEPYEHLSNVKEIVQAFEKKQK
Pdd1_2	ELVFEEIVDKRILDGQTEYLIRFQNVSQPQWVDVGQLIAIKDDVIAYEDKIA
Pdd3	EYEVEKIIKTKYDDQLRTNLYLVKWKGYADHLNTWEPEWNLENSKEILNDFKKKN
Cdl1	LYEVEAIRGHKGNGKNRLYQIKWLGYPENQNTWEPLENLQNILKMVKQYEDSLK
Cdl2	MYEPEYLIDKRVVNGQIEYLVKWVEFSAEESTWEPAENIQVCAFDLIQLFEDSKK
Cdl3_1	NYIVEKIIKKRIKNKKCEYYVKWQGYPSNKNTWEPLSNLENVKDMIYQYEYDLP
Cdl3_2	DHTKIERIFGHDRIGKKSEYLVSYTGIPKPLWVLKHDLPISYYKQYES

Appendix Table S2. Alignment *of P. tetraurelia* CDs.

CD regions were identified by InterProScan https://www.ebi.ac.uk/interpro/search/sequence/), and annotated sequences (InterPro accession: IPR000953) were extracted (Dataset EV1). The CD sequences were aligned using MAFFT v7.520 with "einsi" mode (Katoh & Standley, 2013). Aromatic cage residues are shaded; clasp residues are denoted by an asterisk and polar residues in these positions are marked in bold; clasp residues and the predicted isoelectric point (pI) of the CDs are shown on the right side. *(Div)* denotes CDs with divergent aromatic residues. Related to Figure 1, 2 and Appendix Figure S1.

Name	Chromodomain alignment	Clasp residues	pI
Lace1	**E**YEVESIVEKKFD--EQTKAYLYQIKWKGYPHSQNTWEPIEHL**Q**NPHVKKMVKEFDSTQETTPTKK--	**EQ**	6.34
Lace2	**E**YEVESIIDRRFD--EQAKSYLYQIKWKGYPHSQNTWEPIEHL**Q**NPHVKTMVKEFDSAQEIGSSKK--	**EQ**	5.88
Crn1	LYTVEKVVDKRYDPVSKELQYC--IKWEGYSEADNTWEPLKNL**K**--NVLSEIEEFENQQNNKKQNI--	L**K**	4.85
Crn2	LYIVEKIVDKRYDPLSKELQYC--IKWEGYNEAENTWEPLTNL**K**--NVLSEIEEFENQYRNKKYNK--	L**K**	5.10
Crn3	FFNVEKIVGKRYDPETNELQYC--IKWEGYPDEENTWEPVENL**Q**--NVLSFVQEYEAYQNSTRKKP--	F**Q**	4.55
Cica1	**Q**FDVEFVHGKRYL---NSIEYY--IKWVGYSKTESTWEPETAF**E**-GISNQLITRFERCQNYKNYHT--	**QE**	6.09
Cica2	**Q**FDVEFVHGKRYQ---NSFEYY--IKWVGYSKQESTWEPETAF**E**-GISNQLITRFERCQNYKNYHT--	**QE**	6.09
Cica3	**H**YEVEYVFGKRLDK-GNEVDYA--VKWLGYDKKHHTWEPISSF**S**-AASLLLIGRFERYLAYKNYY---	**HS**	8.16
Cica4	**H**YEVEYVFGKRLDK-GNVVDYA--VKWLGYDKKHHTWEPMSSF**S**-AASLLLIGRFERYLAYKNYY---	**HS**	8.90
Cica5	INQAQFIYGKKLT--KNNYYYA--VKWQGQPISNMSWEKQSNF**T**-EESSYLIRRFET-----------	I**T** *(Div)*	9.40
Cica6	IYDVEFVYGKKMF--KKRTYYA--VKWLGWTIQQMSWEPVSNF**T**-SISSYLVRRLET-----------	I**T**	9.63
Cica7	IYEVEFVYGKKMF--KKQTLYA--VKWLGWAKSQMTWEPFSNF**T**-RISSYLVRRLETRLSYAQYHK--	I**T**	9.93
Fire1	LYEIQYIFGSQIL--DNKMQYC--VKWKNYPKSQRTWEPIENLG-VHNISSVRRFQ------------	LG	9.30
Fire2	LYEIQYIFGSQIL--DNKMQYC--VKWKNYPKSQRTWEPIENLG-AHNISSVRRYQ------------	LG	9.25
Bee	LYEVESIIMKKIE--NQESHYL--VKWKGY--SELTWEPLSSL**Q**--HCQLLVDEFDEQ----------	L**Q**	4.56
May1	**E**YMVEAITNKRVK--NGRTEYE--VKWQGYSDNEKTWEPIENL**Q**--SVMTYVLDFEQSLKQKQNQE--	**EQ**	4.83
May2	**E**YMVEAITNKRVK--NGRTEYE--VKWQGYSDNEKTWEPIENL**Q**--SVMTYVLDFEQSLKQKQPQE--	**EQ**	4.83
May3	**E**YMVEAITNKRVK--NGRTEYE--VKWQGYSENEKTWEPIENL**Q**--SVMTYVLDFEQSLKTKPQEV--	**EQ**	4.86
May4	**E**YMVEAITNKRFK--NGRAEYE--IKWQGYSDNEKTWEPIENL**Q**--TVMTYVLEFEQSLKASGEGS--	**EQ**	4.67
Wasp	LFVVEAIVGMRKN--LSKIEYQ--VKWLGYSKDENTWELQENLV-QNCSDLINQYHQN----------	LV	5.09
Hov1	LFSMEAILDSRTI--NDQLYFL--VKWAGYPISQSTWEKADKVP--ADSNMISE--------------	LP	4.39
Hov2	LFSMEAILDSRTI--NDQLYFL--VKWAGYPISQSTWEKADKVP--ADSNMISE--------------	LP	4.39
Hov3	LYFIEQILDYQQY--NGQKYYL--VKWQGYNNRDCTWEKPEKIP--NLPYFLSEYE------------	LP	4.77
Hov4	LYFIEQILDYKQY--QGQKYYL--VKWQGYNNRDCTWEKPEKIP--NLTQYLNEYE------------	LP	5.15
Hov5	LYFIEQILDYKQY--NGQKYYL--VKWQGYNNRDCTWEKPDKIP--NLTQFLHQFE------------	LP	6.76
Hov6	LYLIDEILDYKQY--NGQKYYL--VKWQGYNNRDCTWEKPEKIP--NLAQFLHQFEEKVKTFGSNF--	LP	6.77
Drg1	MKEPEKIIKKQHI--NGQLKYQ--VKWKGF--DETTWEVEENV**K**--KYKELIED--------------	M**K** *(Div)*	6.57
Drg2	MKEPEKIIKKQHI--NGQLKYQ--VKWKGF--DETTWEVEDNV**K**--KYKELIED--------------	M**K** *(Div)*	6.56
Drg3	MKEPEKIMKKNIL--NGQIKYQ--VKWKGF--DETTWESDETM**K**--KYKELIEDYNYFSLTGERYD--	M**K** *(Div)*	5.38
Drg4	MKEPEKIMKKNIL--NGQIKYQ--VKWKGF--DETTWESDETM**K**--KYKELIEDYNYFSLTGERYD--	M**K** *(Div)*	5.38
Drg5	**Q**RSPEKILQRRNN--NNQLQYL--VKWKGN--EEPTWEFEENI**R**NIIQNLPVNEQELYSNQNDKQVKQ	**QR** *(Div)*	6.45
Drg6	**Q**RSPEKILSKRNN--NGQLQYL--IKWKNH--EEPTWEFEEYI**R**NQIQNLLTSELEQKTHQNEKQV--	**QR** *(Div)*	6.94
Drg7	PRSPERILKKRLI--NNQIQYL--MKWKGT--DQTTWEYEQNV**R**RSILEEFLQNQQSYQEQKQKNL--	P**R** *(Div)*	9.70
Drg8	PRSPERILKKRIL--NNQTQYL--MKWKGS--EQTTWEYEENV**R**RSMLEEFLQNQQSSQEQKQKNL--	P**R** *(Div)*	9.54
	* *		

Appendix Table S3. Sequence of codon-optimized May1-His for *E. coli* protein expression.

Related to Figure 2.

Codon-optimized May1-His
ATGAGCGGTAGCCCGGTGAGCGTTAGCAGCCAGGAAGAGTACATGGTGGAGGCGATCACCAACAAG<br>CGTGTTAAAAACGGTCGTACCGAGTACGAAGTGAAGTGGCAAGGCTATAGCGACAACGAGAAAACC<br>TGGGAGCCGATTGAAAACCTGCAGAGCGTGATGACCTACGTTCTGGATTTCGAACAAAGCCTGAAG<br>CAGAAACAAAACCAGGAAGTGGGTGAAGGCAACTATGACGATGGTGACAGCGCGGATGAGATCCTG<br>CAGATTCGTAAGGACAACGATGGCCAAAACCTGCTGTTTCAAGTTAGCTGGAAGCAGAAGAACAAC<br>CTGGCGCCGAAAGTGAGCTGGGTTAACCAAAACACCCTGAAAATGCACAACCCGGAAATCCTGATT<br>GACTATCTGCTGAAGAAAATCAAGTGGCCGAACAACAAACACCACCACCACCACCACTGA

Black: Codon-optimized May1 (PTET.51.1.G1380122); Blue: His tag; Red: Stop codon

Appendix Table S4. Histone peptides used in this study.

The following commercially available histone peptides were used for the peptide pulldown. Note that they are not *Paramecium* peptide sequences, and that while the trimethylated residue and most other residues surrounding it are conserved, not all residues are identical. Related to Figure 2.

Peptide name	***Company***	***Catalog #***	***Sequence***
Histone H3.1 aa1-43 Peptide, Biotinylated	EpiCypher	EP120065	ARTKQTARKSTGGKAPRKQLATKAARKSAPATGGVKKPHRYRP-K-(Biotin)-CONH2
Histone H3K9me3 Peptide, Biotinylated	Active Motif	81047	ARTKQTAR-Kme3-STGGKAPRKQLA-GGYK(Biotin)-NH2
Histone H3K27me3 Peptide, Biotinylated	Active Motif	81052	ATKAAR-Kme3-SAPSTGGVKKPHRYRPG-GGK(Biotin)-NH2

References

Arnaiz O, Meyer E, Sperling L (2020) ParameciumDB 2019: integrating genomic data across the genus for functional and evolutionary biology. *Nucleic Acids Research* 48: D599-D605

Arnaiz O, Van Dijk E, Betermier M, Lhuillier-Akakpo M, de Vanssay A, Duharcourt S, Sallet E, Gouzy J, Sperling L (2017) Improved methods and resources for paramecium genomics: transcription units, gene annotation and gene expression. *BMC Genomics* 18

Katoh K, Standley DM (2013) MAFFT Multiple Sequence Alignment Software Version 7: Improvements in Performance and Usability. *Molecular Biology and Evolution* 30: 772-780

Wang C, Solberg T, Maurer-Alcalá XX, Swart EC, Gao F, Nowacki M (2022) A small RNA-guided PRC2 complex eliminates DNA as an extreme form of transposon silencing. *Cell Reports* 40

Wiley EA, Horrell S, Yoshino A, Schornak CC, Bagnani C, Chalker DL (2018) Diversification of HP1-like Chromo Domain Proteins in Tetrahymena thermophila. *Journal of Eukaryotic Microbiology* 65: 104-116
