# Supplementary figures and images for: Heterochromatin-dependent transcription links the PRC2 complex to small RNA-mediated DNA elimination
Source: EMBO Rep. 2024 Nov 29;26(1):273–96. doi: 10.1038/s44319-024-00332-1 (PMC11723920; doi:10.1038/s44319-024-00332-1)

Figure 2G

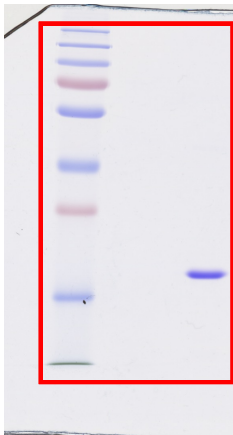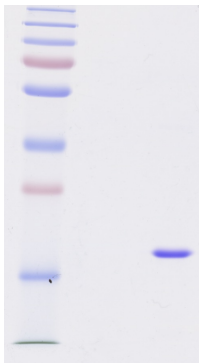

May1-His

Supplement: Supplementary file 6 — Source data Fig. 2 [file 44319_2024_332_MOESM6_ESM.zip › Figure 2/2G/Coomassie.pdf]

Figure 2H

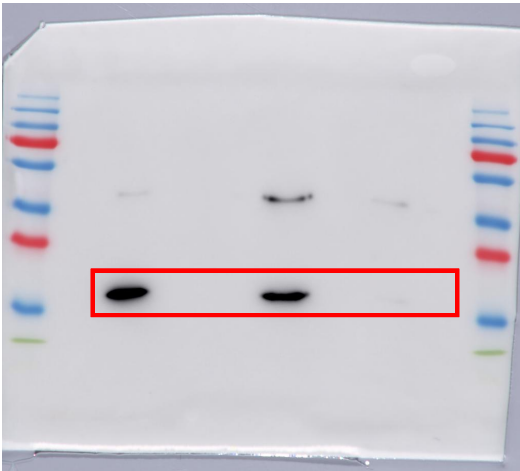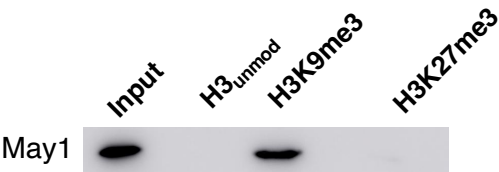

Supplement: Supplementary file 6 — Source data Fig. 2 [file 44319_2024_332_MOESM6_ESM.zip › Figure 2/2H/Western blot.pdf]

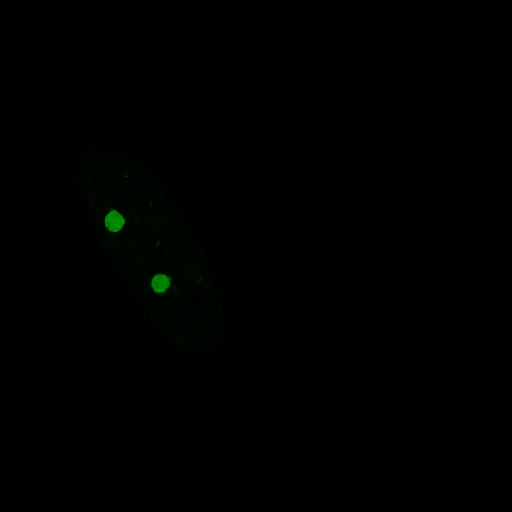

Supplement: Supplementary file 6 — Source data Fig. 2 [file 44319_2024_332_MOESM6_ESM.zip › Figure 2/2C/GFP.tif]

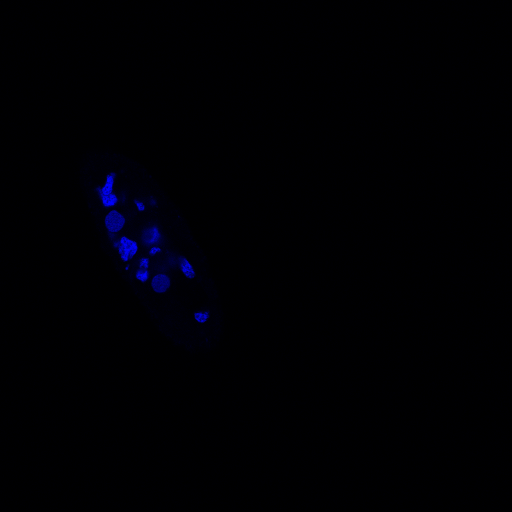

Supplement: Supplementary file 6 — Source data Fig. 2 [file 44319_2024_332_MOESM6_ESM.zip › Figure 2/2C/DAPI.tif]

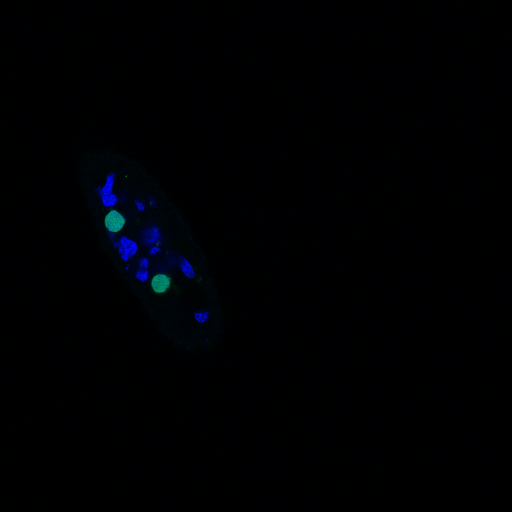

Supplement: Supplementary file 6 — Source data Fig. 2 [file 44319_2024_332_MOESM6_ESM.zip › Figure 2/2C/Merge.tif]

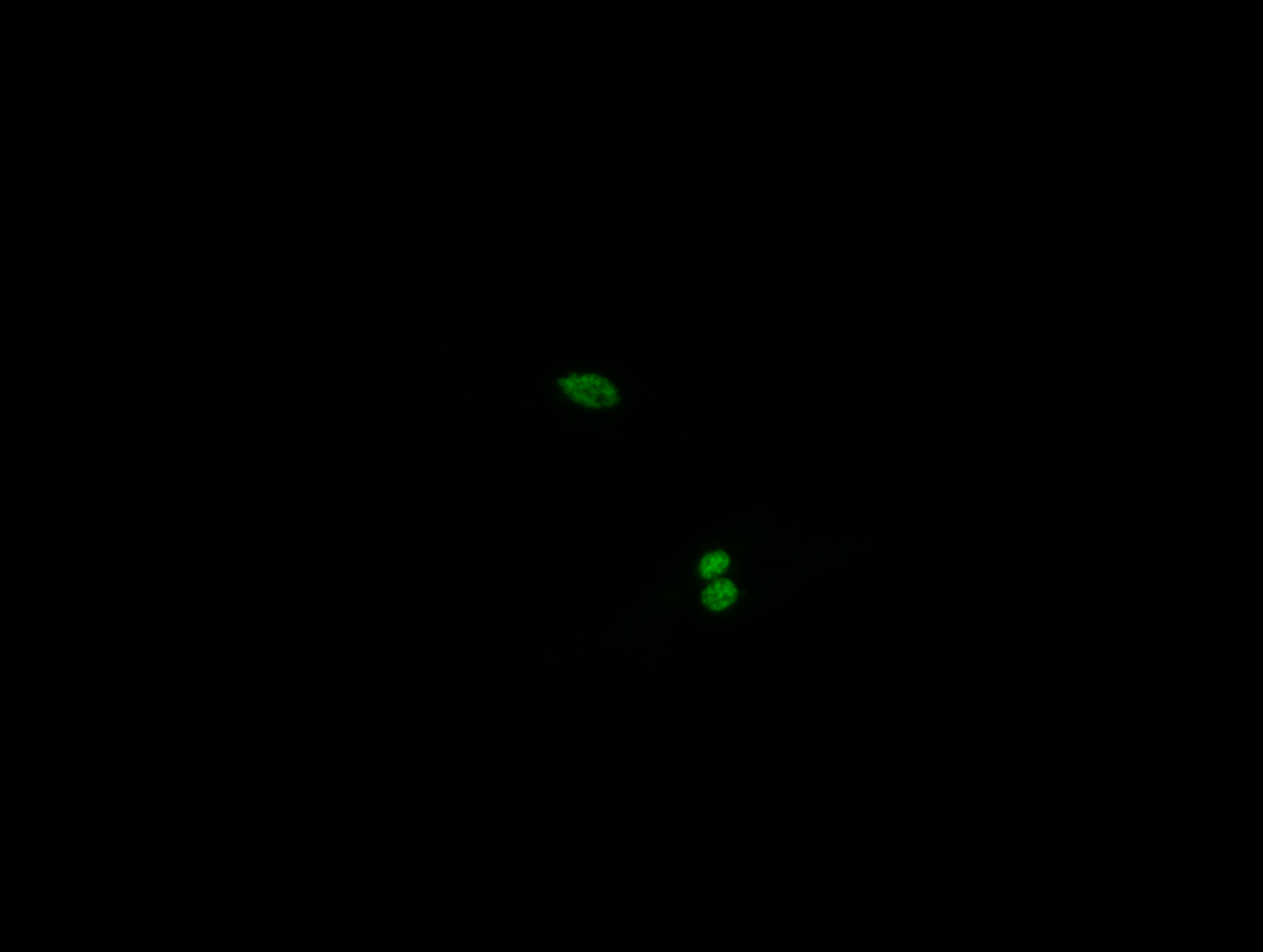

Supplement: Supplementary file 6 — Source data Fig. 2 [file 44319_2024_332_MOESM6_ESM.zip › Figure 2/2D/GFP.tif]

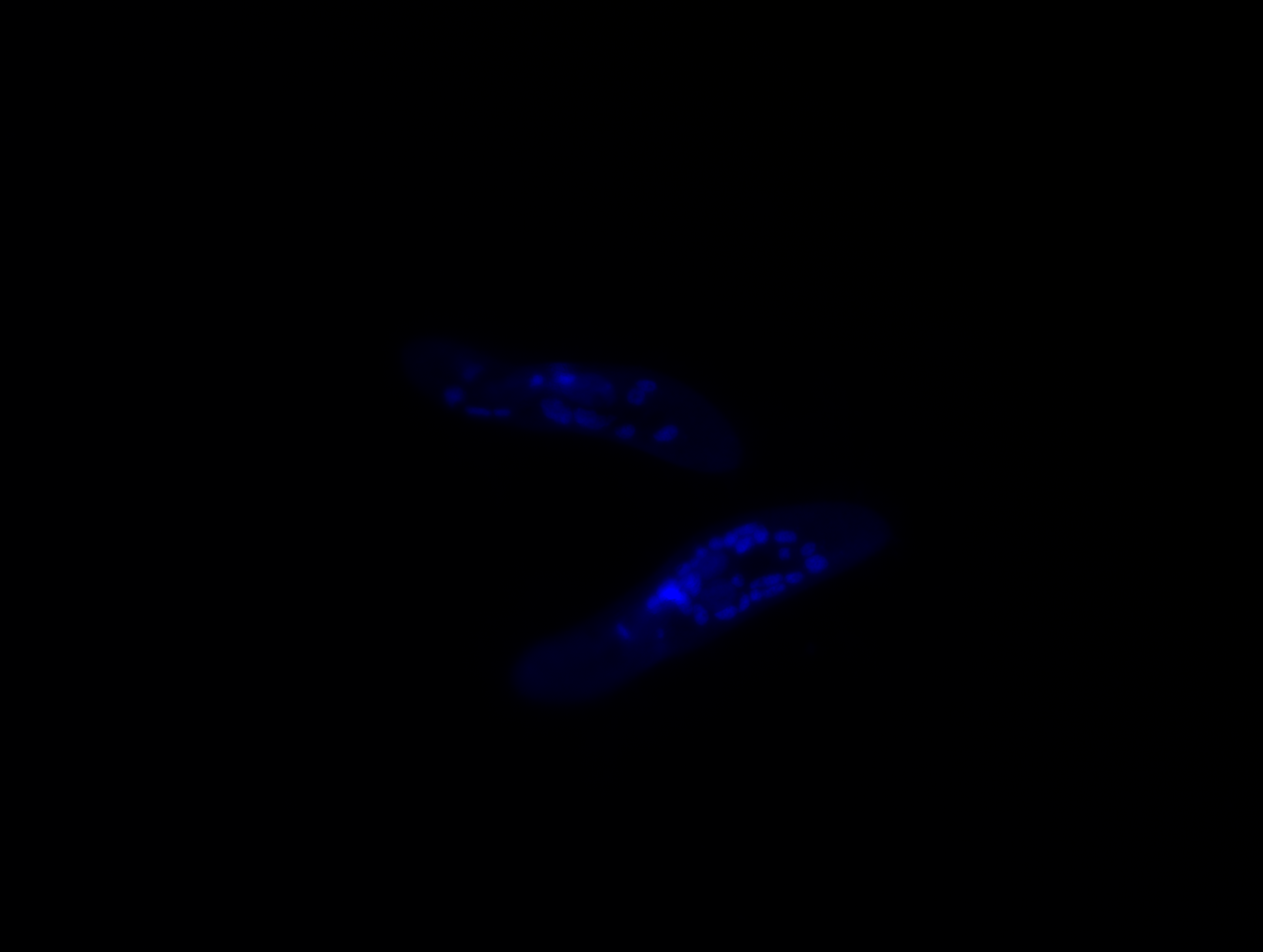

Supplement: Supplementary file 6 — Source data Fig. 2 [file 44319_2024_332_MOESM6_ESM.zip › Figure 2/2D/DAPI.tif]

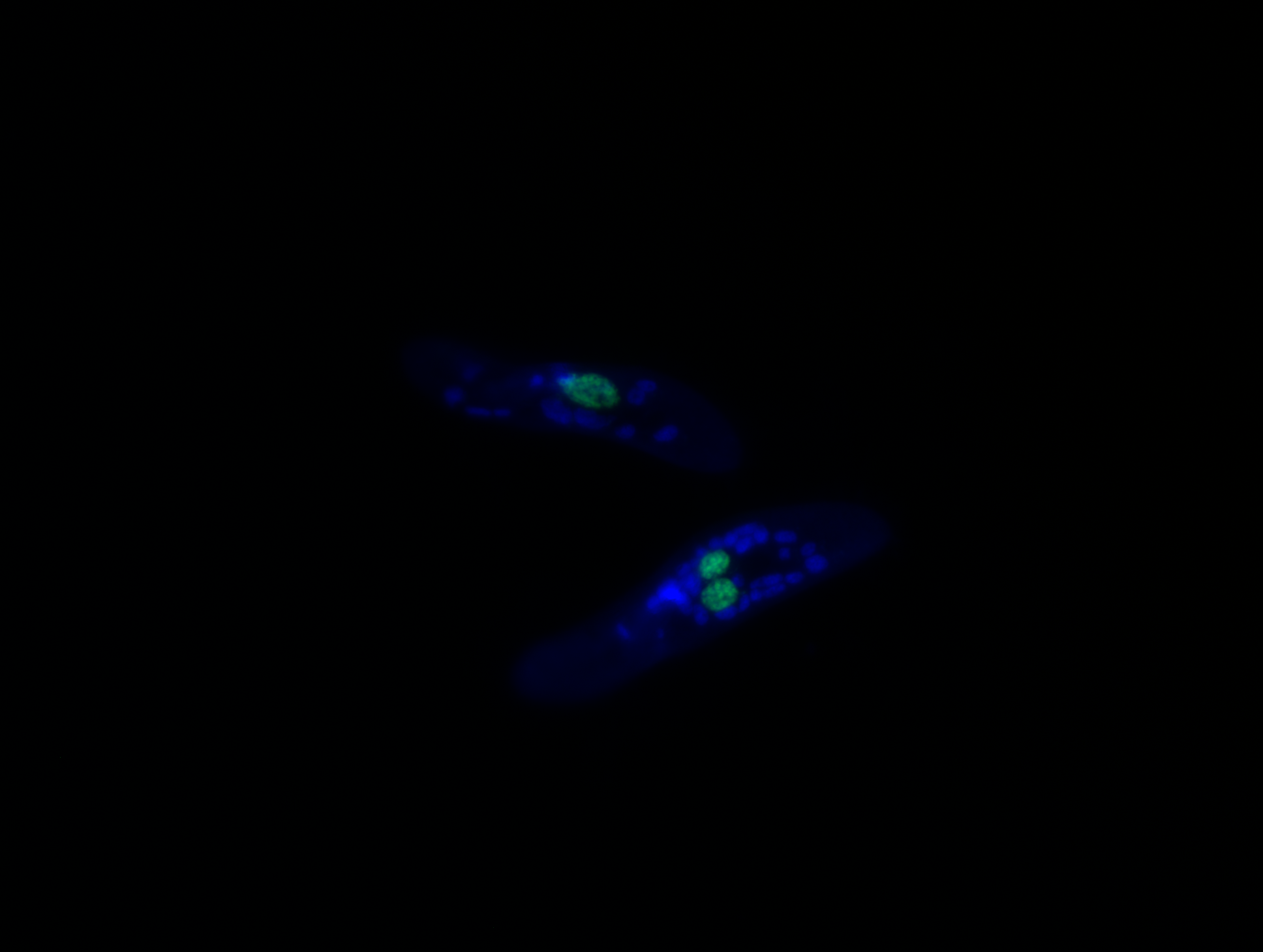

Supplement: Supplementary file 6 — Source data Fig. 2 [file 44319_2024_332_MOESM6_ESM.zip › Figure 2/2D/Merge.tif]

Figure 4E

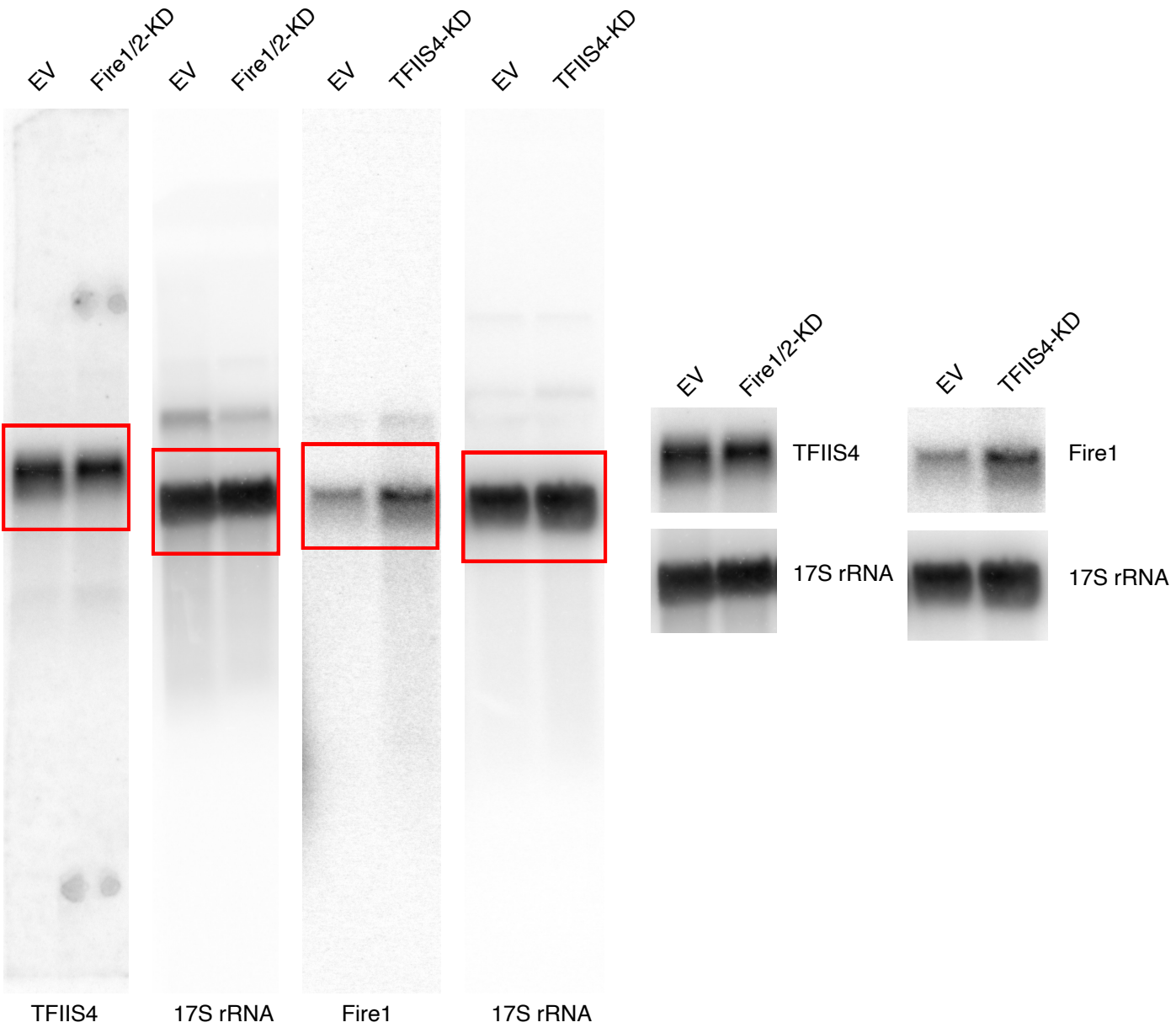

Supplement: Supplementary file 8 — Source data Fig. 4 [file 44319_2024_332_MOESM8_ESM.zip › Figure 4/4E/Northern blots.pdf]

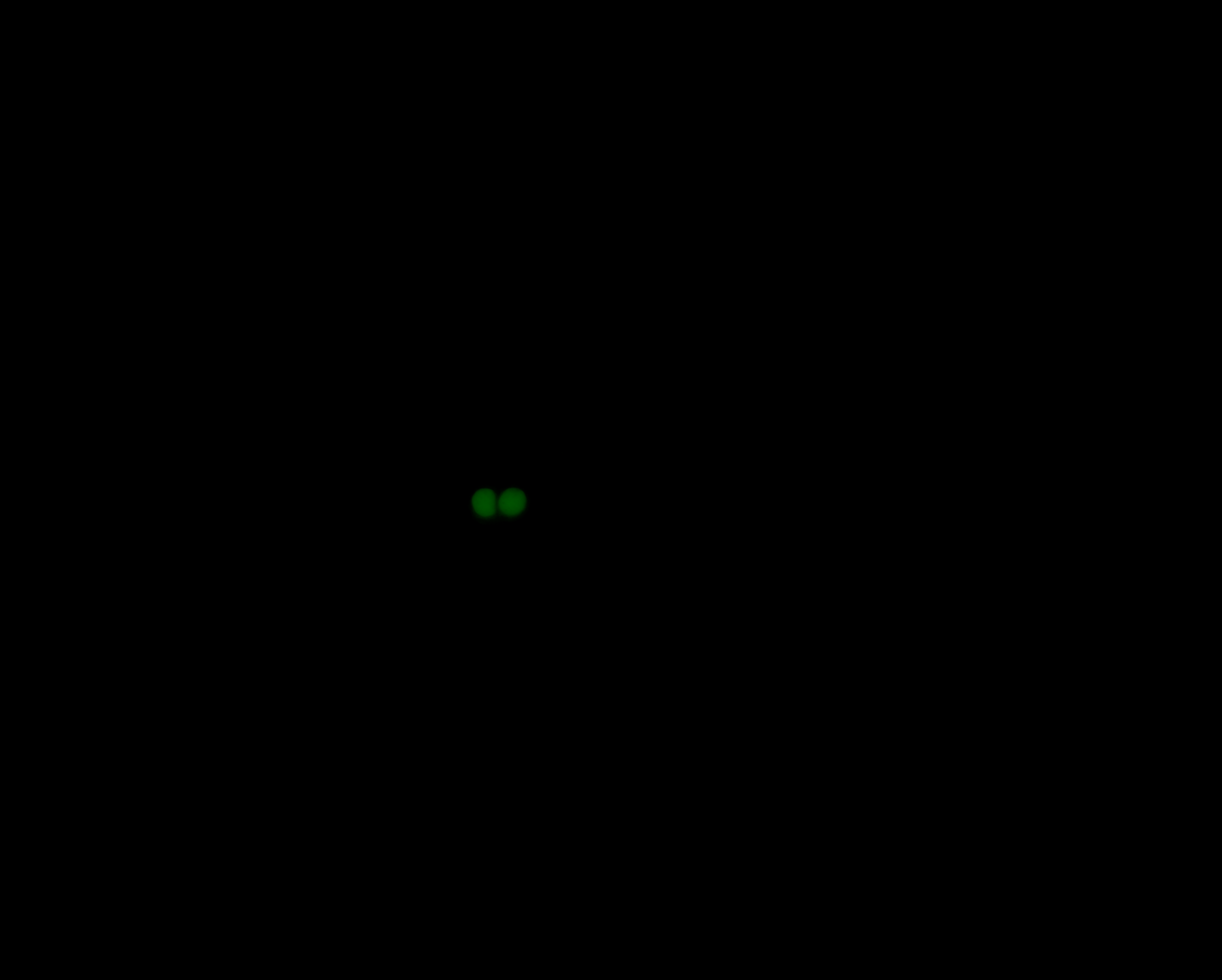

Supplement: Supplementary file 8 — Source data Fig. 4 [file 44319_2024_332_MOESM8_ESM.zip › Figure 4/4D/Fire1-GFP (bottom)/EV/GFP.tif]

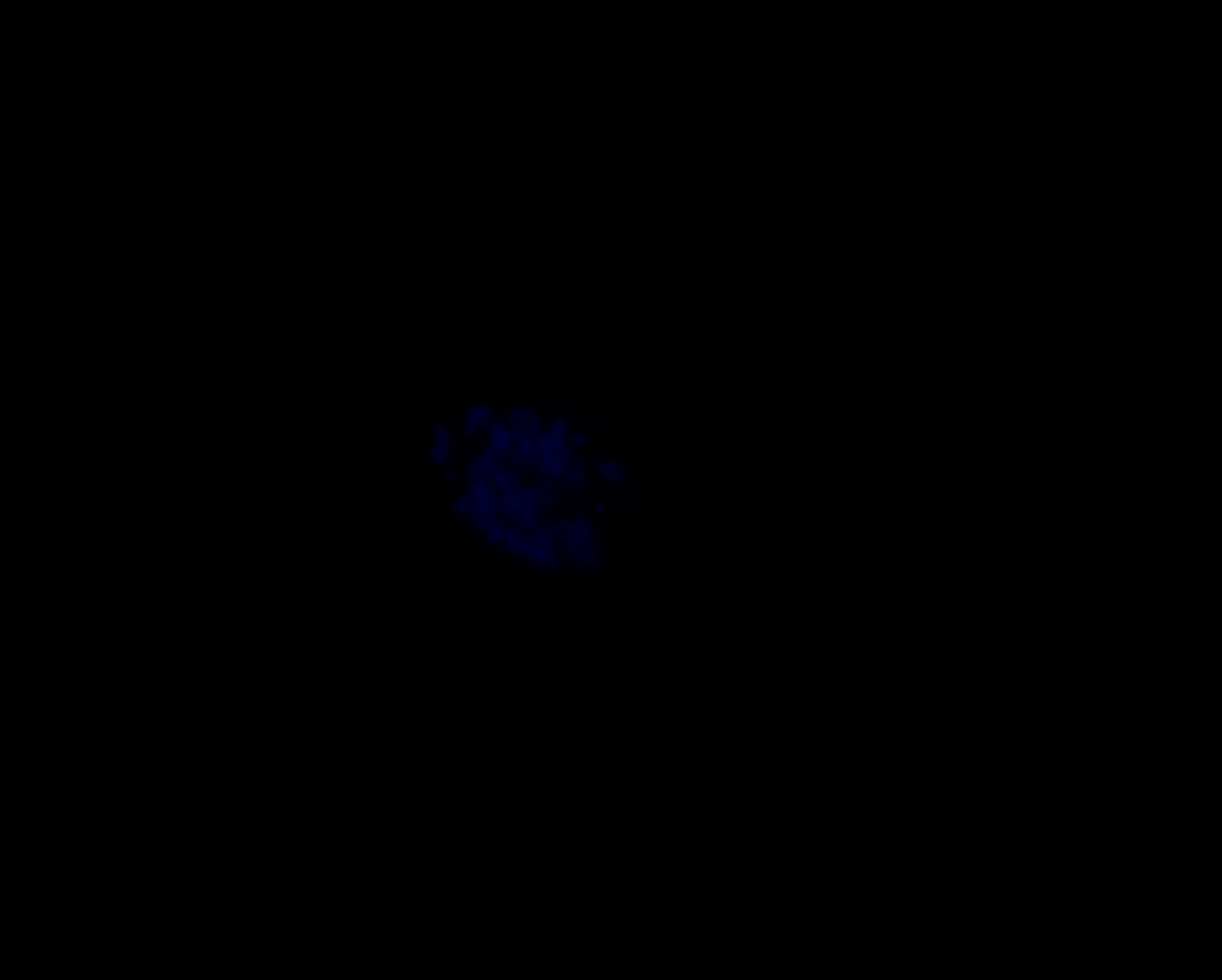

Supplement: Supplementary file 8 — Source data Fig. 4 [file 44319_2024_332_MOESM8_ESM.zip › Figure 4/4D/Fire1-GFP (bottom)/EV/DAPI.tif]

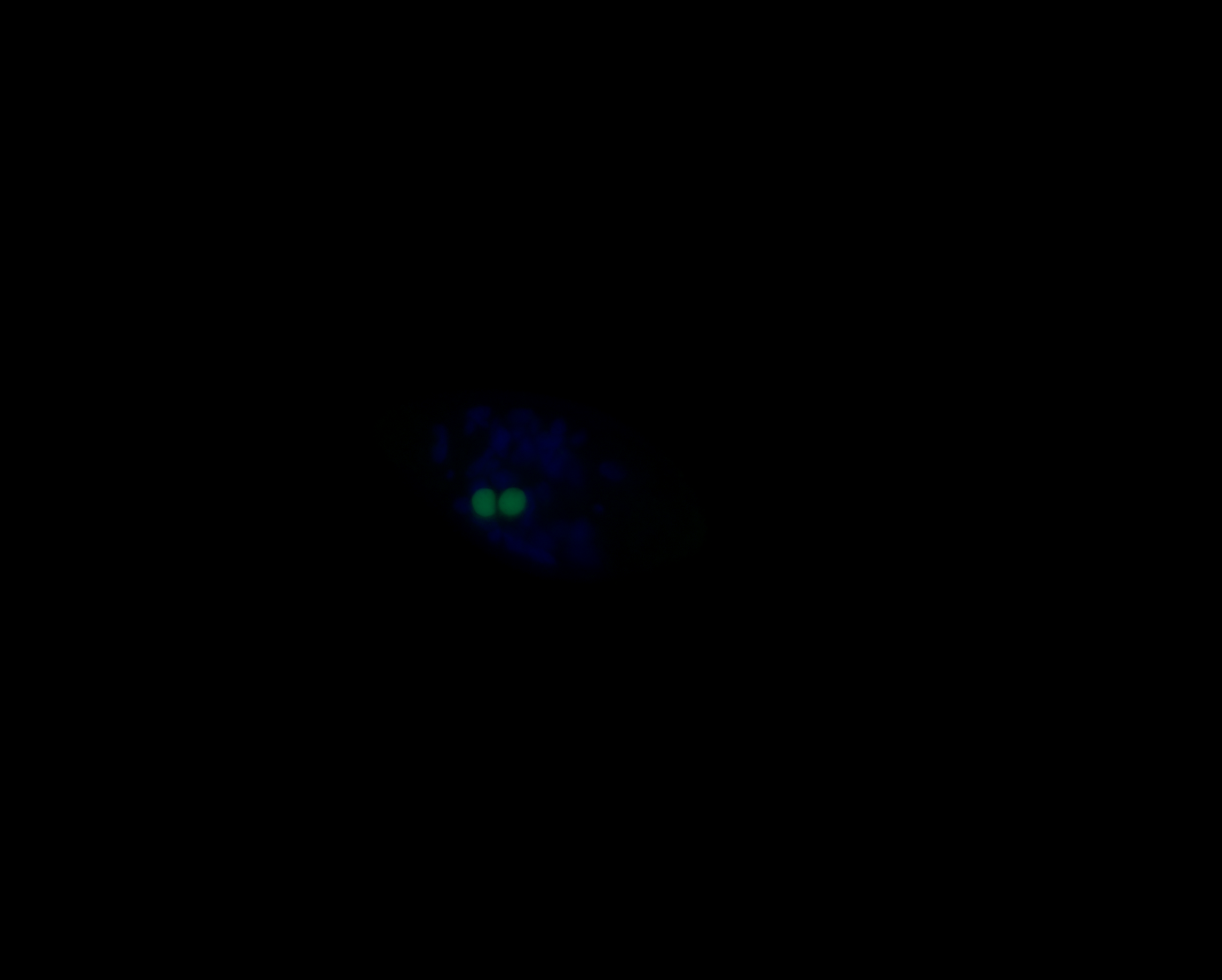

Supplement: Supplementary file 8 — Source data Fig. 4 [file 44319_2024_332_MOESM8_ESM.zip › Figure 4/4D/Fire1-GFP (bottom)/EV/Merge.tif]

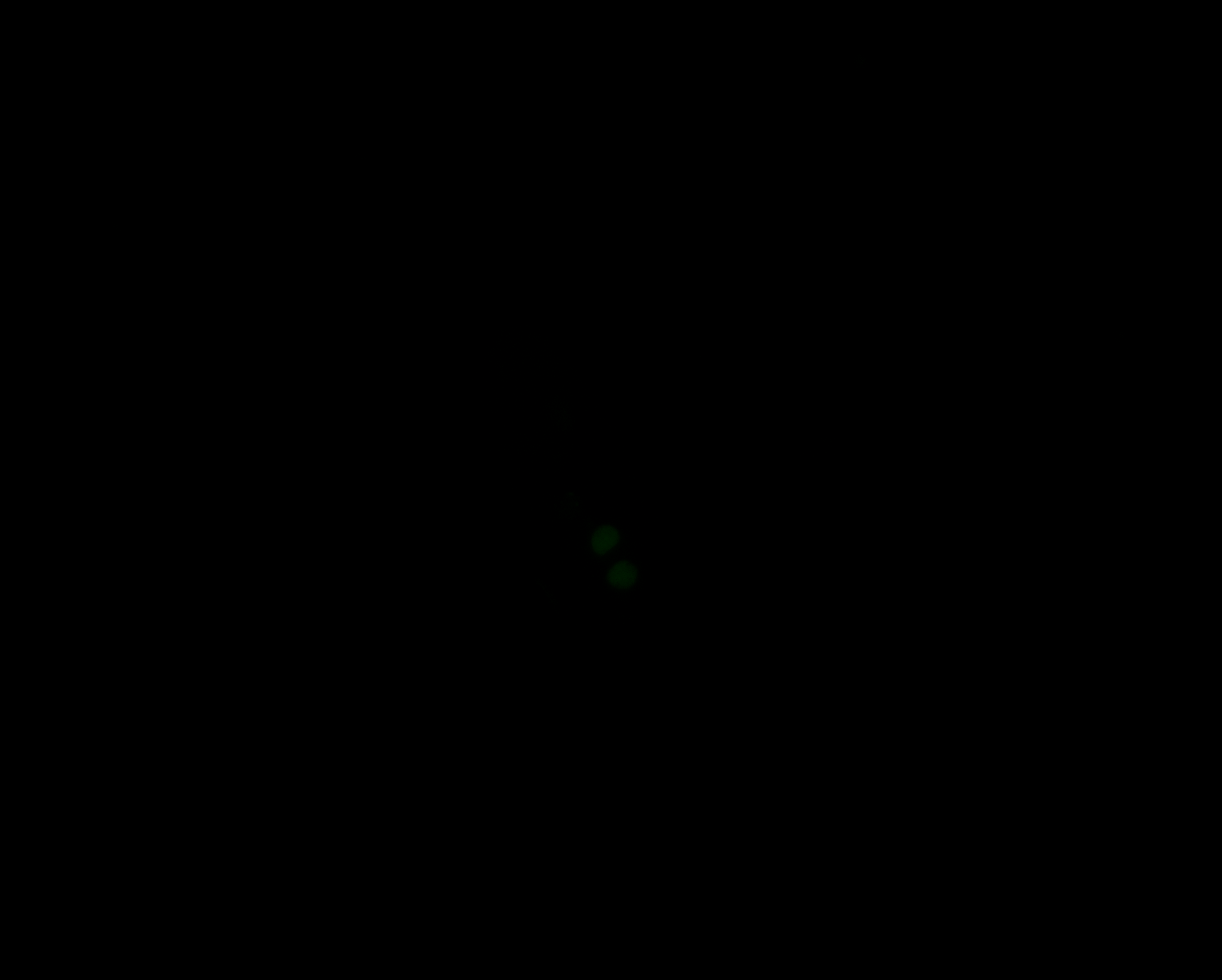

Supplement: Supplementary file 8 — Source data Fig. 4 [file 44319_2024_332_MOESM8_ESM.zip › Figure 4/4D/Fire1-GFP (bottom)/TFIIS4-KD/GFP.tif]

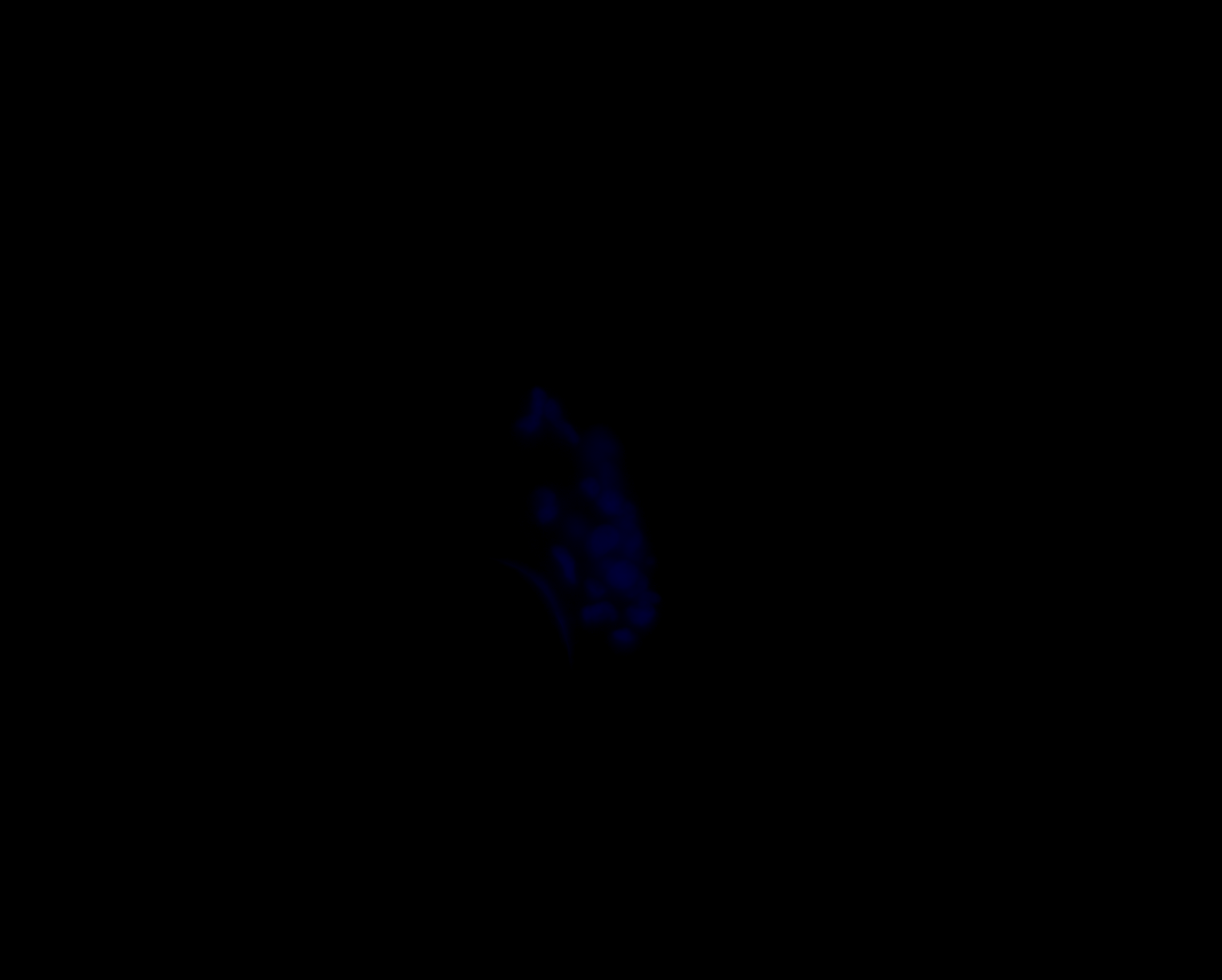

Supplement: Supplementary file 8 — Source data Fig. 4 [file 44319_2024_332_MOESM8_ESM.zip › Figure 4/4D/Fire1-GFP (bottom)/TFIIS4-KD/DAPI.tif]

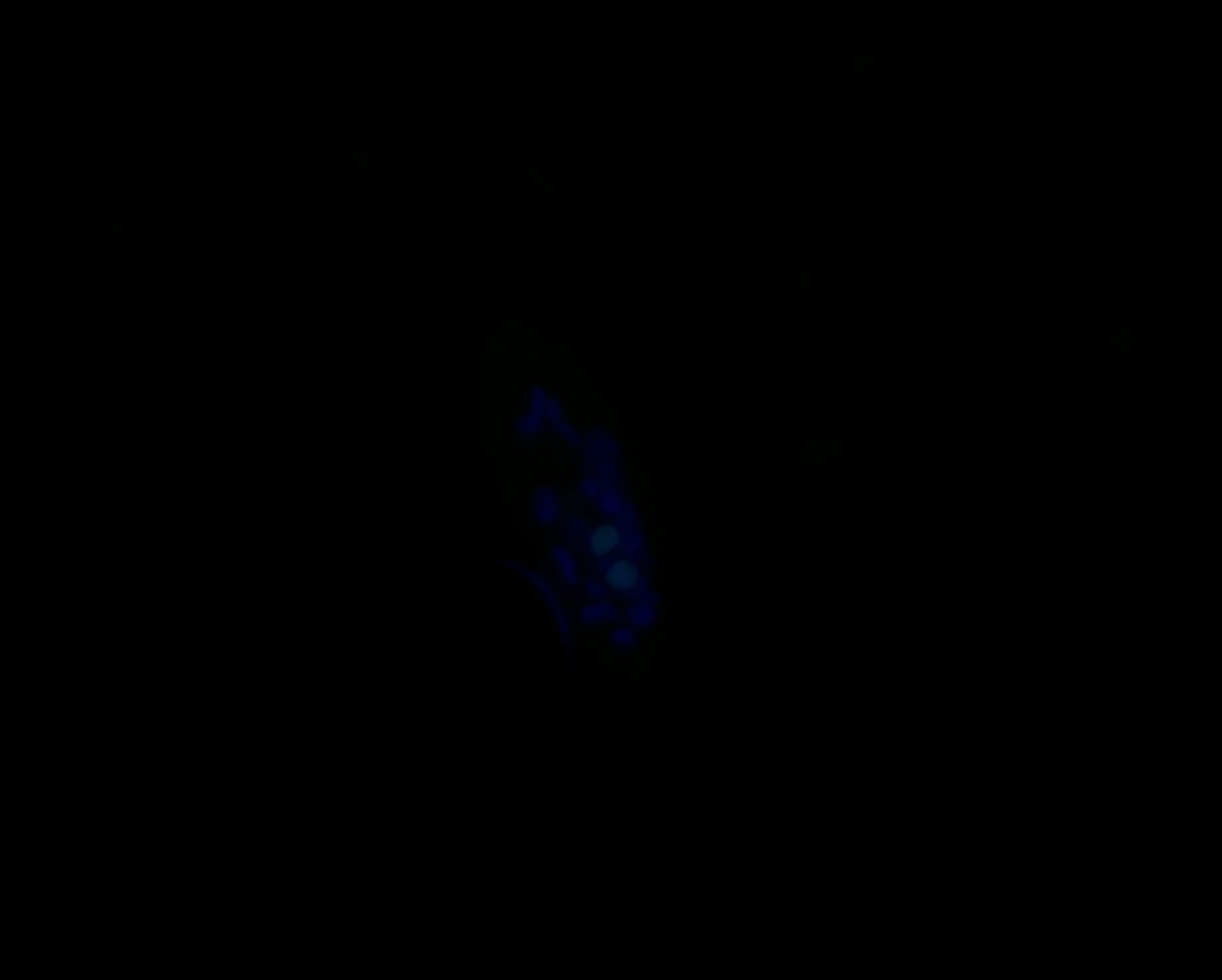

Supplement: Supplementary file 8 — Source data Fig. 4 [file 44319_2024_332_MOESM8_ESM.zip › Figure 4/4D/Fire1-GFP (bottom)/TFIIS4-KD/Merge.tif]

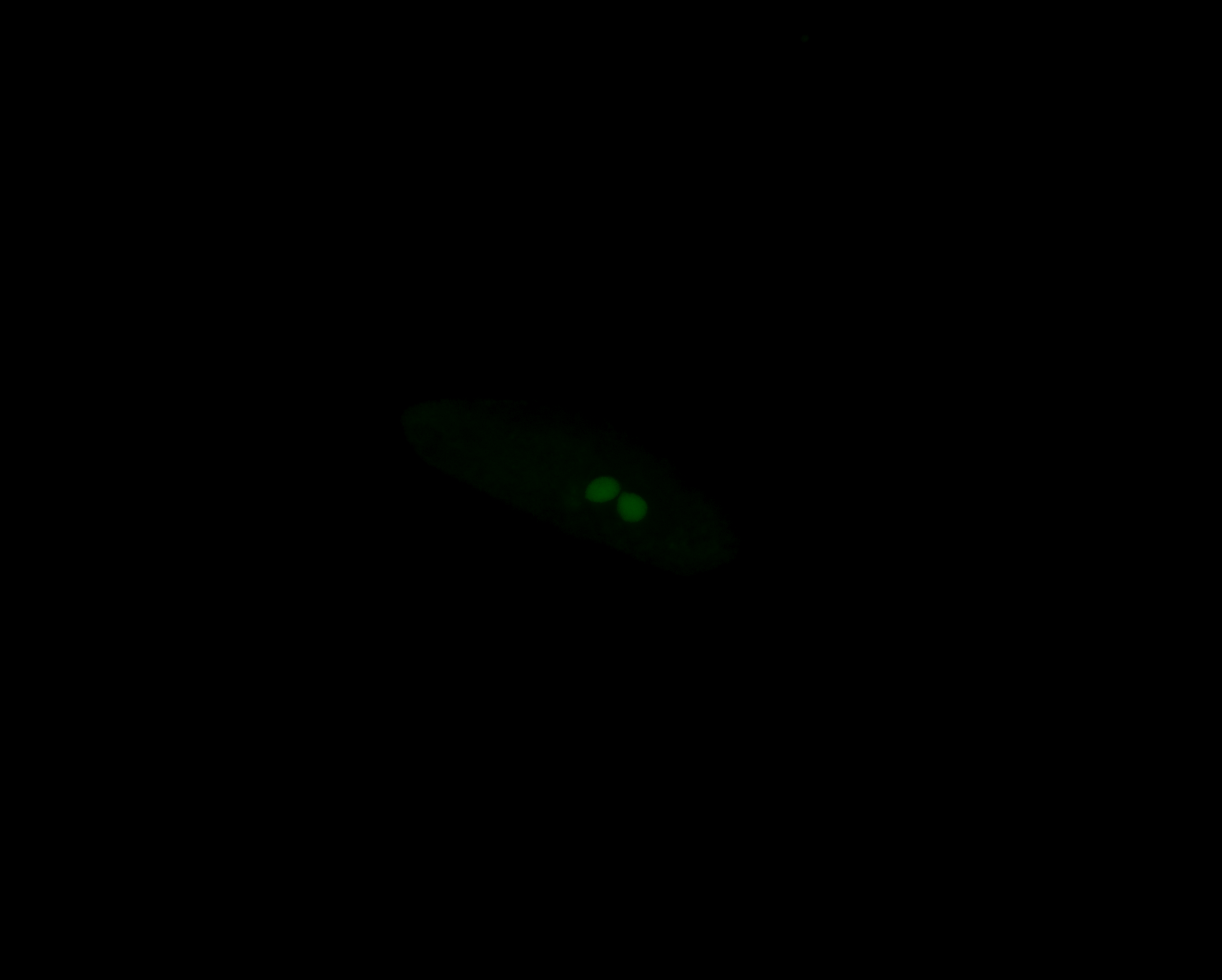

Supplement: Supplementary file 8 — Source data Fig. 4 [file 44319_2024_332_MOESM8_ESM.zip › Figure 4/4D/TFIIS4-GFP (top)/EV/GFP.tif]

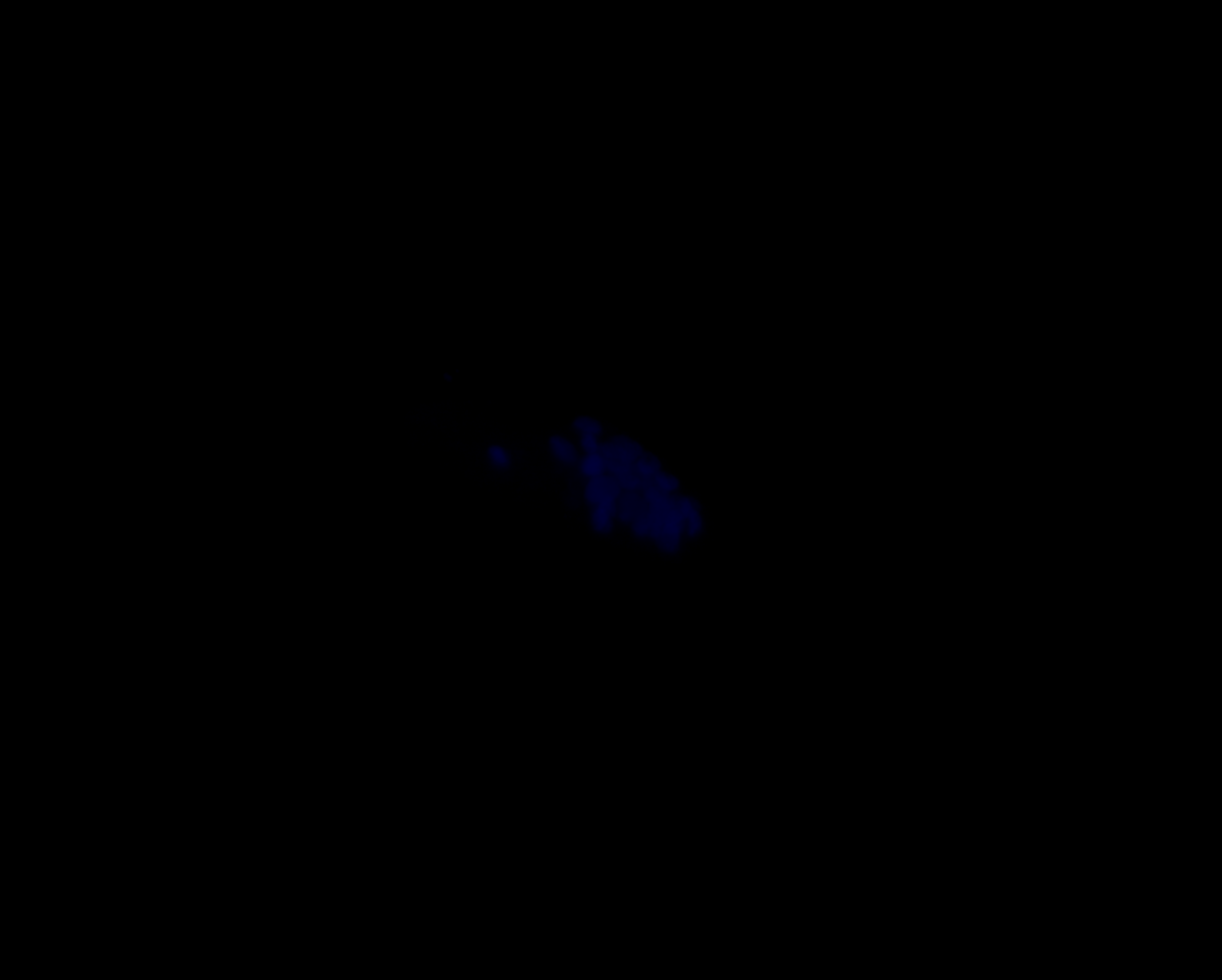

Supplement: Supplementary file 8 — Source data Fig. 4 [file 44319_2024_332_MOESM8_ESM.zip › Figure 4/4D/TFIIS4-GFP (top)/EV/DAPI.tif]

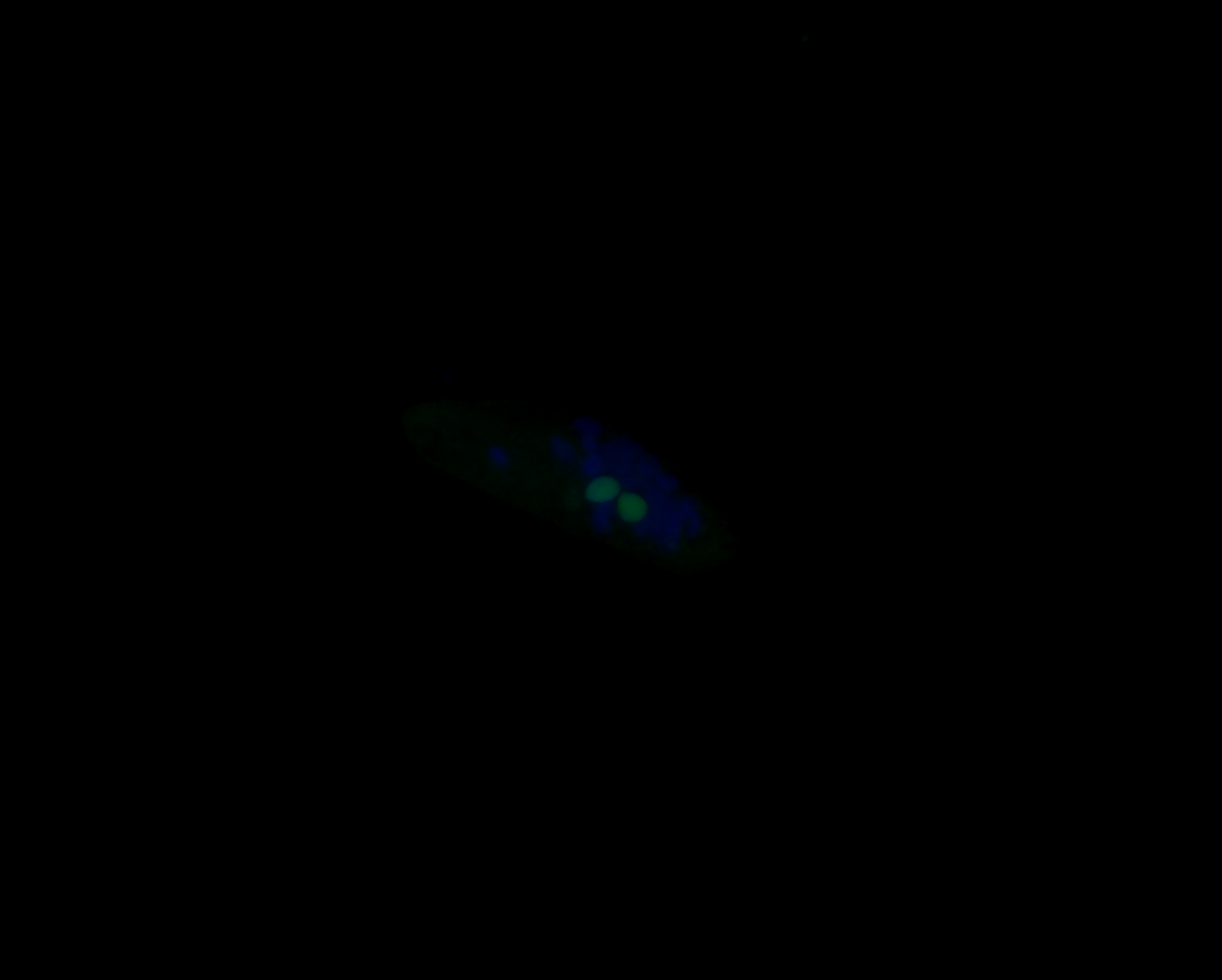

Supplement: Supplementary file 8 — Source data Fig. 4 [file 44319_2024_332_MOESM8_ESM.zip › Figure 4/4D/TFIIS4-GFP (top)/EV/Merge.tif]

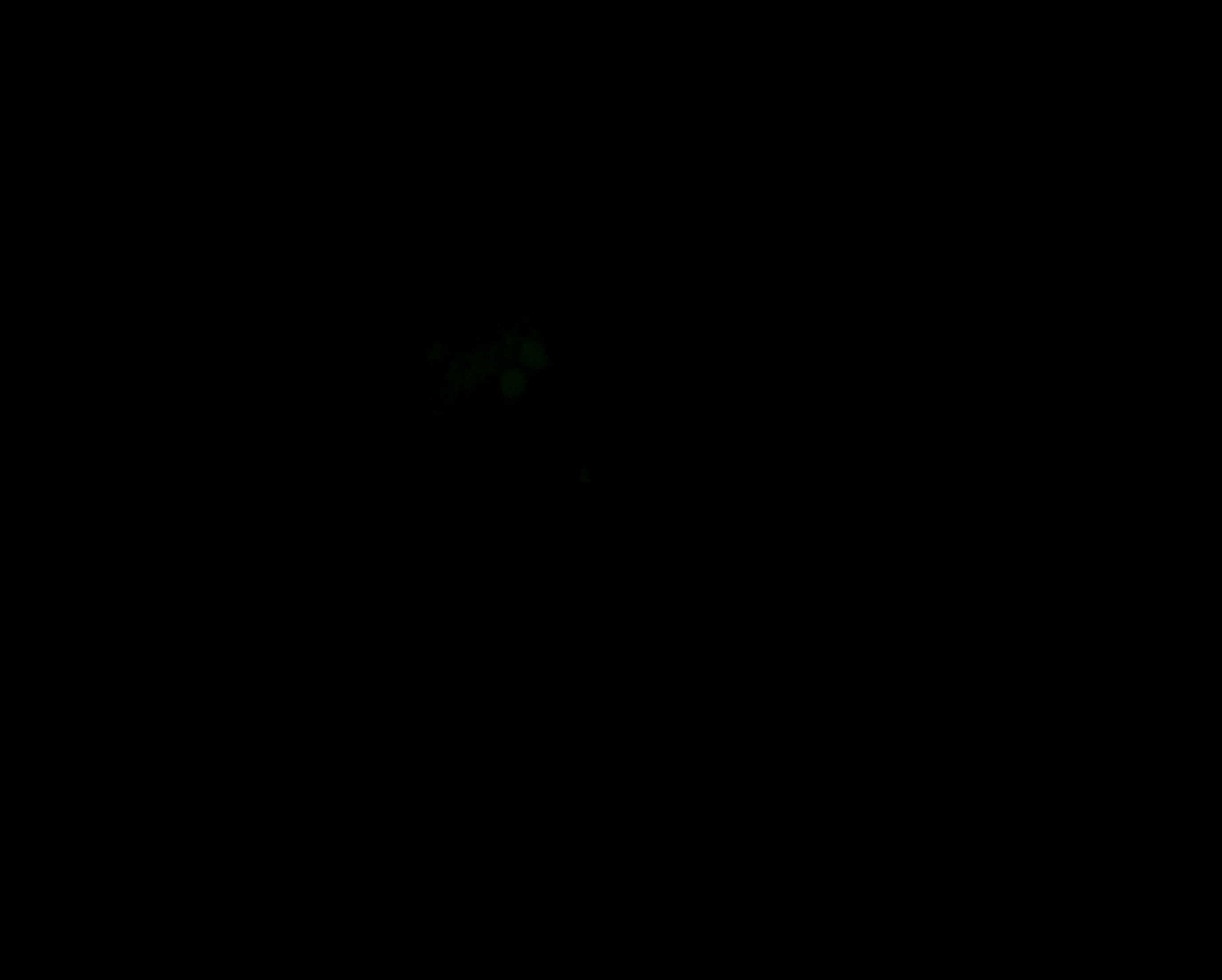

Supplement: Supplementary file 8 — Source data Fig. 4 [file 44319_2024_332_MOESM8_ESM.zip › Figure 4/4D/TFIIS4-GFP (top)/Fire1:2-KD/GFP.tif]

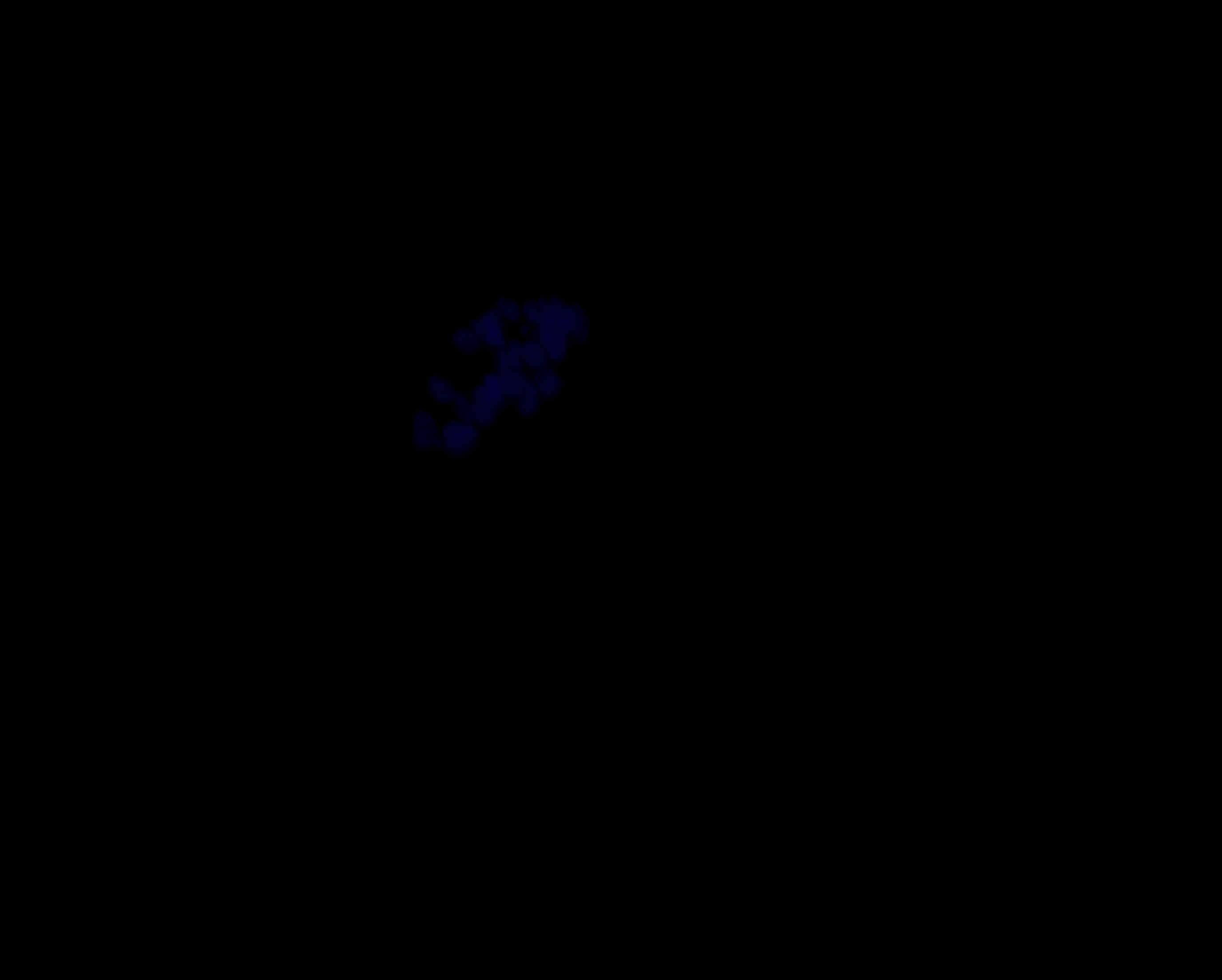

Supplement: Supplementary file 8 — Source data Fig. 4 [file 44319_2024_332_MOESM8_ESM.zip › Figure 4/4D/TFIIS4-GFP (top)/Fire1:2-KD/DAPI.tif]

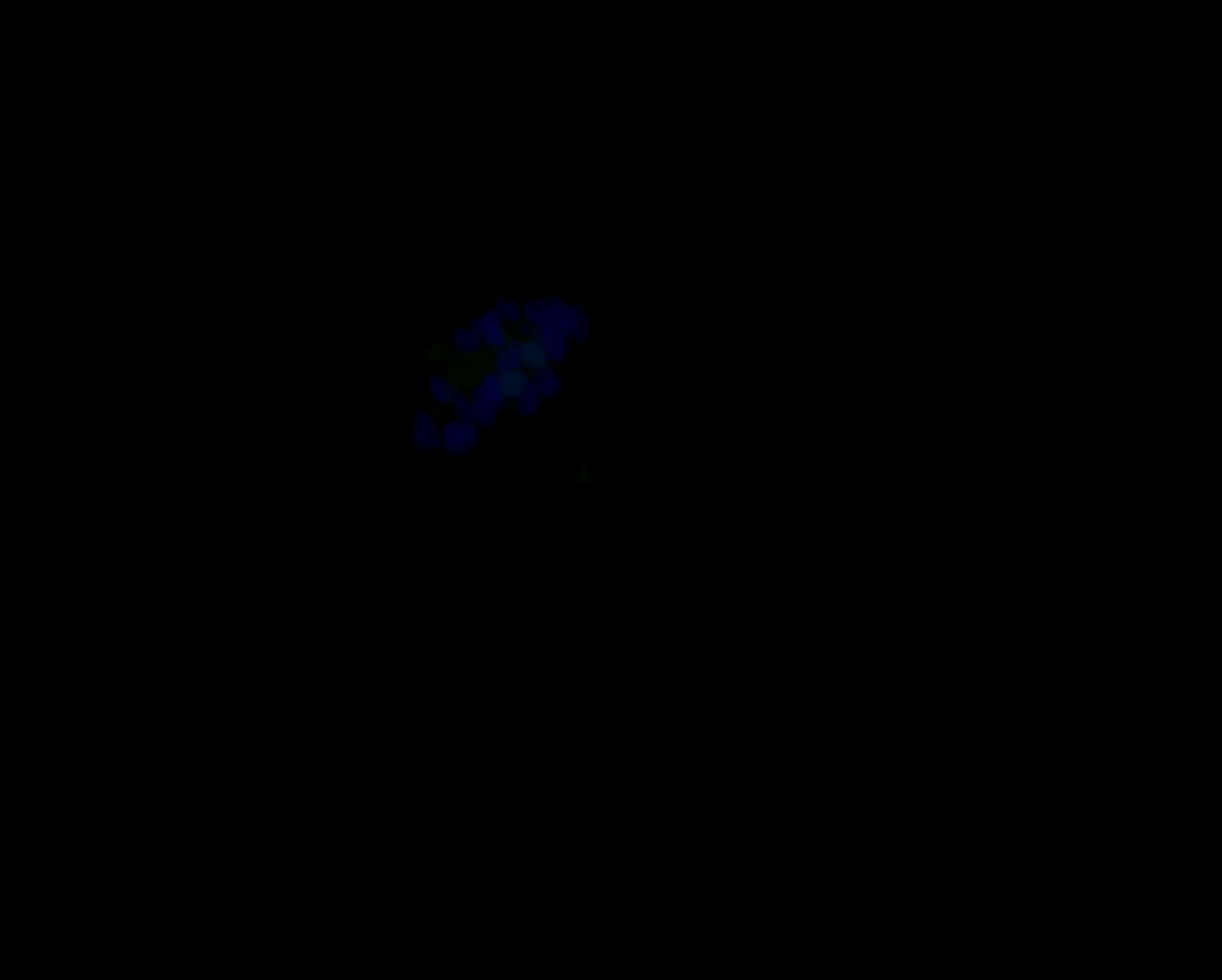

Supplement: Supplementary file 8 — Source data Fig. 4 [file 44319_2024_332_MOESM8_ESM.zip › Figure 4/4D/TFIIS4-GFP (top)/Fire1:2-KD/Merge.tif]
