# Supplementary material for: Heterochromatin-dependent transcription links the PRC2 complex to small RNA-mediated DNA elimination
Source: EMBO Rep. 2024 Nov 29;26(1):273–96. doi: 10.1038/s44319-024-00332-1 (PMC11723920; doi:10.1038/s44319-024-00332-1)
Supplement: Supplementary file 10 — Expanded View Figures [file 44319_2024_332_MOESM10_ESM.pdf]

Expanded View Figures

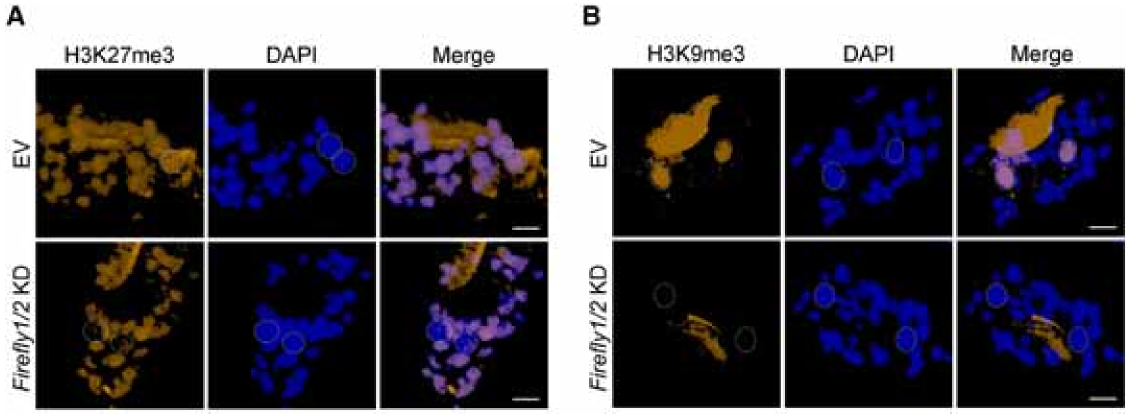

Figure EV1. Fire1/2-KD affects the deposition of H3K27me3 and H3K9me3 in new MACs.

(**A**) Immunofluorescence of H3K27me3 at the late stage of development in EV and Fire1/2-silenced cells. (**B**) Immunofluorescence of H3K9me3 at the late stage of development in EV and Fire1/2-silenced cells. Dotted circles denote new MACs. Scale bars: 10 µm.

© The Author(s)

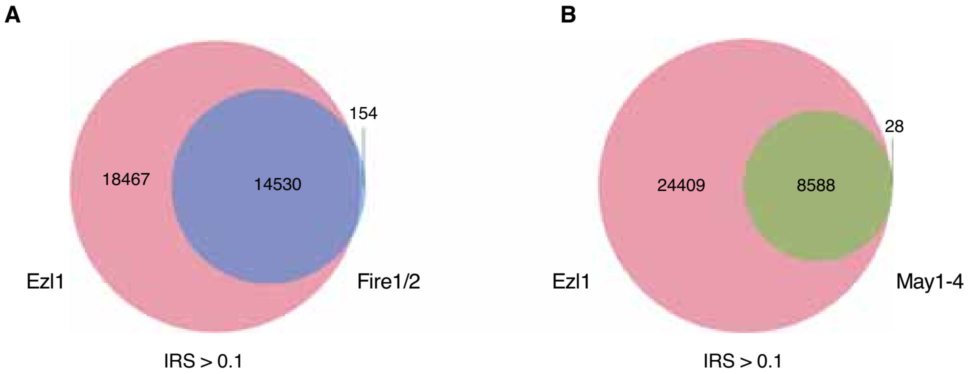

Figure EV2. Shared IES retention between Ezl1, Fire1/2 and May1-4 silencing.

(**A**) Venn diagram depicting shared IES retention between Ezl1 and Fire1/2 silencing (IRS > 0.1). (**B**) Venn diagram depicting shared IES retention between Ezl1 and May1-4 silencing (IRS > 0.1).

 © The Author(s)

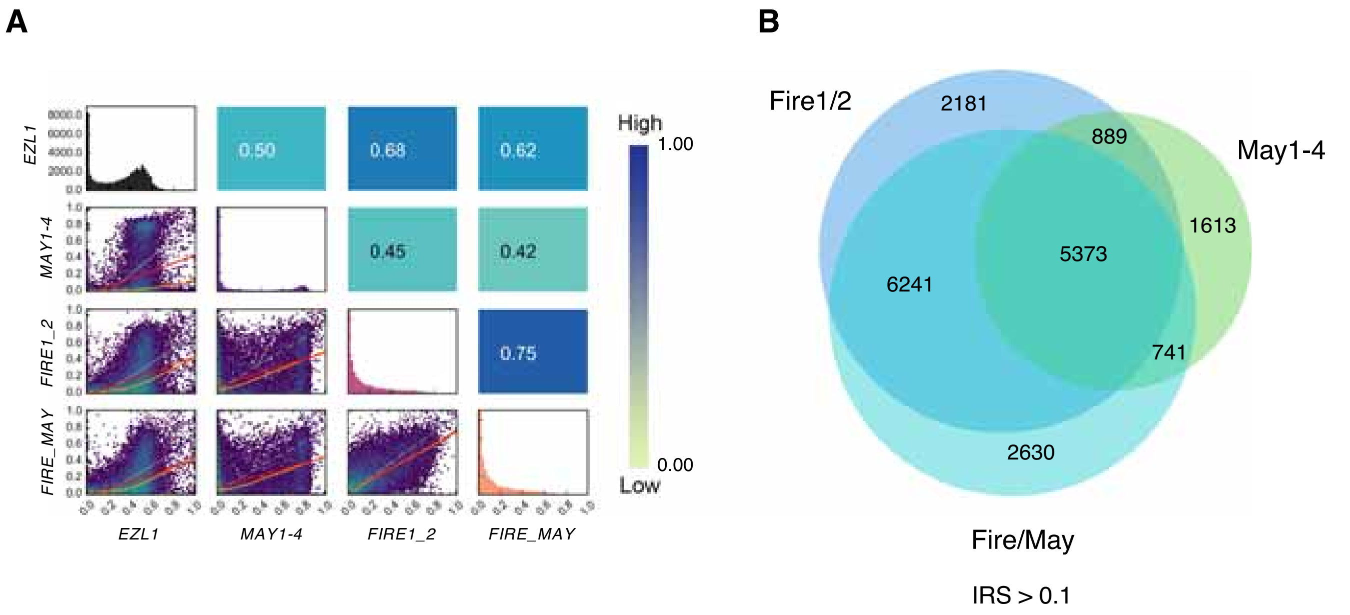

Figure EV3. Correlation and shared IES retention between May1-4, Fire1/2 and Fire/May-KD.

(**A**) Correlation plots calculated by hexagonal binning of IES retention scores generated using After_ParTIES (Swart et al, 2017) and the IES retention scores provided in Dataset EV2. Pearson's correlation coefficients are given above each subgraph. Red lines are for ordinary least-squares (OLS) regression, orange lines for LOWESS, and gray lines for orthogonal distance regression (ODR). From light green to dark blue, the correlation is stronger. (**B**) Venn diagram depicting shared IES retention between Fire1/2, May1-4 and Fire1/2/May1-4 ("Fire/May") silencing (IRS > 0.1).

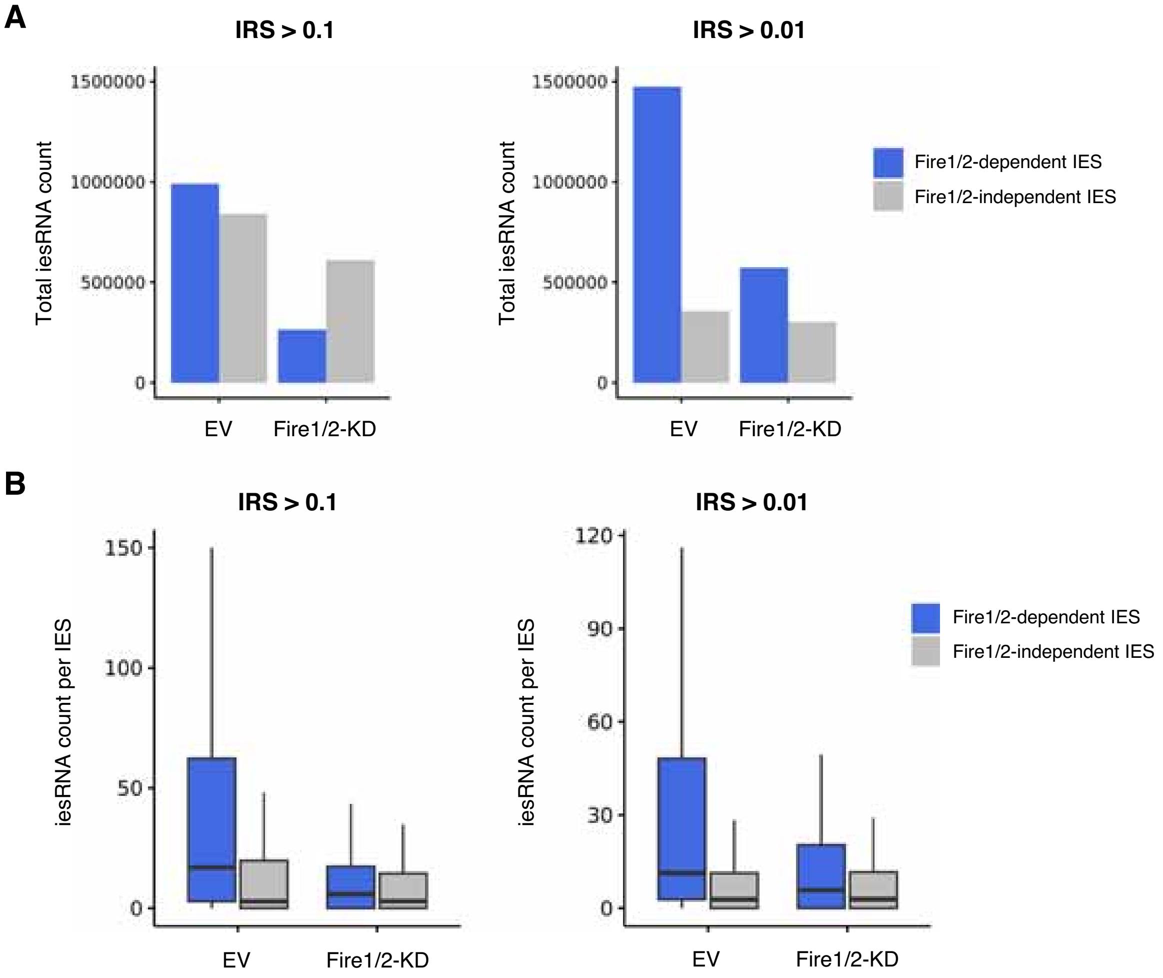

Figure EV4. iesRNAs produced from Fire1/2-dependent and independent IESs.

(**A**) Total iesRNA counts corresponding to Fire1/2-dependent or independent IESs, with a retention score cutoff of 0.1 (left) or 0.01 (right) to denote Fire1/2-dependent IESs. (**B**) iesRNA count per IES for Fire1/2-dependent or independent IESs, with a retention score cutoff of 0.1 (left) or 0.01 (right) to denote Fire1/2-dependent IESs. The bold line denotes the median, the lower and upper hinges the 25th and 75th percentiles and the whiskers extend to the largest and smallest values no larger than 1.5 x inter-quartile range (IQR). Outliers were omitted for better visualization. Number of IESs (n) from left to right: IRS > 0.1, 14684, 30243, 14684, 30243; IRS > 0.01, 26840, 18087, 26840, 18087. For all plots, 21 to 24 and 26 to 30 nt sRNA with perfect IES-matching sequences were selected as iesRNAs, and the reads were normalized to the 23 nt siRNAs mapped to the backbone of the L4440 vector.

 © The Author(s)
